# Supplementary material for: Predictability and parallelism in the contemporary evolution of hybrid genomes
Source: PLoS Genet. 2022 Jan 27;18(1):e1009914. doi: 10.1371/journal.pgen.1009914 (PMC8794199; doi:10.1371/journal.pgen.1009914)
Supplement: S1 Table — (DOCX) [file pgen.1009914.s002.docx]

**S1 Table.** Correlations between minor parent ancestry (*X. birchmanni* ancestry) and recombination rate in Santa Cruz and Huextetitla hybrid populations at different non-overlapping window sizes.

| Population | Spearman’s correlation between minor ancestry and recombination rate | | |
| --- | --- | --- | --- |
|  | **50 kb** | **100 kb** | **250 kb** |
| Santa Cruz | *ρ* = 0.40  p < 10^-325^ | *ρ* = 0.44  p = 10^-321^ | *ρ* = 0.51  p = 10^-180^ |
| Huextetitla | *ρ* = 0.37  p < 10^-325^ | *ρ* = 0.42  p = 10^-283^ | *ρ* = 0.50  p = 10^-173^ |
