# Supplementary material for: Predictability and parallelism in the contemporary evolution of hybrid genomes
Source: PLoS Genet. 2022 Jan 27;18(1):e1009914. doi: 10.1371/journal.pgen.1009914 (PMC8794199; doi:10.1371/journal.pgen.1009914)
Supplement: S2 Table — See Methods and Text K in S1 File for more details on these analyses. AIMs–ancestry informative sites; Rec–recombination. In the mask short tracts analysis, we removed ancestry tracts shorter than 0.004 cM for minor parent ancestry tracts and 0.035 cM for major parent ancestry tracts, based on the reasoning that these unusually short tracts compared to expectations given the age of the hybrid population [34] might represent switch errors. In the thinned physical distance analysis, one window was retained every 500 kb. In the exclude inversions category, we removed chromosomes 21 and 24 which have large inversions between X. birchmanni and X. cortezi. (DOCX) [file pgen.1009914.s003.docx]

**S2 Table.** Correlations between minor parent ancestry (*X. birchmanni* ancestry) and recombination rate in Santa Cruz and Huextetitla hybrid populations using different approaches to control for variation in power to infer local ancestry or to infer recombination rate. See Methods and Text K in S1 File for more details on these analyses. AIMs – ancestry informative sites; Rec – recombination. In the mask short tracts analysis, we removed ancestry tracts shorter than 0.004 cM for minor parent ancestry tracts and 0.035 cM for major parent ancestry tracts, based on the reasoning that these unusually short tracts compared to expectations given the age of the hybrid population [35] might represent switch errors. In the thinned physical distance analysis, one window was retained every 500 kb. In the exclude inversions category, we removed chromosomes 21 and 24 which have large inversions between *X. birchmanni* and *X. cortezi*.

| Population | Additional Analysis | Spearman’s correlation between minor parent ancestry and recombination rate | | |
| --- | --- | --- | --- | --- |
|  |  | **50 kb** | **100 kb** | **250 kb** |
| Santa Cruz | thinned AIMs | *ρ* = 0.39  p < 10^-325^ | *ρ* = 0.43  p = 10^-303^ | *ρ* = 0.49  p = 10^-167^ |
|  | thinned Rec Map | *ρ* = 0.38  p < 10^-325^ | *ρ* = 0.43  p = 10^-296^ | *ρ* = 0.50  p = 10^-173^ |
|  | thinned AIMs & thinned Rec Map | *ρ* = 0.38  p < 10^-325^ | *ρ* = 0.41  p = 10^-278^ | *ρ* = 0.49  p = 10^-161^ |
|  | mask short tracts | *ρ* = 0.40  p < 10^-325^ | *ρ* = 0.44  p = 10^-323^ | *ρ* = 0.51  p = 10^-182^ |
|  | thinned physical distance | *ρ* = 0.41  p = 10^-56^ | *ρ* = 0.47  p = 10^-72^ | *ρ* = 0.53  p = 10^-96^ |
|  | exclude inversions | *ρ* = 0.40  p < 10^-325^ | *ρ* = 0.44  p = 10^-301^ | *ρ* = 0.51  p = 10^-170^ |
| Huextetitla | thinned AIMs | *ρ* = 0.36  p < 10^-325^ | *ρ* = 0.40  p = 10^-257^ | *ρ* = 0.58  p = 10^-156^ |
|  | thinned Rec Map | *ρ* = 0.35  p < 10^-325^ | *ρ* = 0.40  p = 10^-262^ | *ρ* = 0.49  p = 10^-165^ |
|  | thinned AIMs & thinned Rec Map | *ρ* = 0.34  p < 10^-325^ | *ρ* = 0.38  p = 10^-238^ | *ρ* = 0.47  p <= 10^-149^ |
