# Supplementary material for: Predictability and parallelism in the contemporary evolution of hybrid genomes
Source: PLoS Genet. 2022 Jan 27;18(1):e1009914. doi: 10.1371/journal.pgen.1009914 (PMC8794199; doi:10.1371/journal.pgen.1009914)
Supplement: S3 Table — See Methods for details. (DOCX) [file pgen.1009914.s004.docx]

**S3 Table.** Correlations between minor parent ancestry (*X. birchmanni* ancestry) and recombination rate in Santa Cruz and Huextetitla hybrid populations using a F_2_ crossover map to estimate recombination rate. See Methods for details.

| Population | Additional Analysis | Spearman’s correlation between minor parent ancestry and crossover rate per window | |
| --- | --- | --- | --- |
|  |  | 1 Mb | 5 Mb |
| Santa Cruz | Recombination rate from observed crossovers in F_2_s | *ρ* = 0.64  p = 10^-79^ | *ρ* = 0.69  p = 10^-22^ |
| Huextetitla |  | *ρ* = 0.63  p = 10^-76^ | *ρ* = 0.64  p = 10^-18^ |
