# Supplementary material for: Predictability and parallelism in the contemporary evolution of hybrid genomes
Source: PLoS Genet. 2022 Jan 27;18(1):e1009914. doi: 10.1371/journal.pgen.1009914 (PMC8794199; doi:10.1371/journal.pgen.1009914)
Supplement: S4 Table — 75% of ancestry tracts found in Santa Cruz and Huextetitla are 0.1 cM or larger. (DOCX) [file pgen.1009914.s005.docx]

**S4 Table.** Correlations between minor parent ancestry (*X. birchmanni* ancestry) and the number of coding and conserved basepairs in a range of genetic non-overlapping window sizes. 75% of ancestry tracts found in Santa Cruz and Huextetitla are 0.1 cM or larger.

| Population | Spearman’s partial correlation with minor parent ancestry | | | | | | | |
| --- | --- | --- | --- | --- | --- | --- | --- | --- |
|  | **0.1 cM** | | **0.25 cM** | | **0.5 cM** | | **1 cM** | |
|  | **Coding** | **Conserved** | **Coding** | **Conserved** | **Coding** | **Conserved** | **Coding** | **Conserved** |
| Santa Cruz | *ρ* =0.04  p = 10^-8^ | *ρ* = -0.17  p = 10^-96^ | *ρ* =0.06  p = 10^-6^ | *ρ* = -0.14  p= 10^-54^ | *ρ* =0.05  p= 10^-3^ | *ρ* = -0.18  p= 10^-31^ | *ρ* =0.05  p=0.025 | *ρ* = -0.22  p= 10^-19^ |
| Huextetitla | *ρ* =0.06  p =10^-11^ | *ρ* = -0.14  p = 10^-64^ | *ρ* =0.07  p= 10^-7^ | *ρ* = -0.16  p= 10^-37^ | *ρ* =0.07  p= 10^-4^ | *ρ* = -0.22  p= 10^-24^ | *ρ* =0.06  p= 10^-3^ | *ρ* = -0.19  p= 10^-15^ |
