# Supplementary material for: Predictability and parallelism in the contemporary evolution of hybrid genomes
Source: PLoS Genet. 2022 Jan 27;18(1):e1009914. doi: 10.1371/journal.pgen.1009914 (PMC8794199; doi:10.1371/journal.pgen.1009914)
Supplement: S5 Table — (DOCX) [file pgen.1009914.s006.docx]

**S5 Table.** Analysis of the correlation between minor parent ancestry and linked coding and conserved basepairs in 0.25 cM non-overlapping windows, excluding all regions with structural rearrangements between *X. birchmanni*, *X. malinche*, or *X. cortezi*.

| Population | Spearman’s correlation with minor parent ancestry | |
| --- | --- | --- |
|  | **0.25 cM** | |
|  | **Coding** | **Conserved** |
| Santa Cruz | *ρ* =-0.21  p = 10^-59^ | *ρ* = -0.28  p= 10^-106^ |
| Huextetitla | *ρ* =-0.15  p= 10^-29^ | *ρ* = -0.22  p= 10^-61^ |
