# Supplementary material for: Predictability and parallelism in the contemporary evolution of hybrid genomes
Source: PLoS Genet. 2022 Jan 27;18(1):e1009914. doi: 10.1371/journal.pgen.1009914 (PMC8794199; doi:10.1371/journal.pgen.1009914)
Supplement: S6 Table — Ancestry was summarized using the results of an HMM run on a set of thinned input ancestry informative sites (see Methods). Minor parent ancestry is reduced in regions with a higher number of both synonymous and nonsynonymous substitutions between X. birchmanni and X. malinche. This may be driven by a correlation between coding substitutions and regions with a high number of linked coding basepairs (S7 Table). (DOCX) [file pgen.1009914.s007.docx]

**S6 Table.** Relationship between minor parent ancestry (*X. birchmanni* ancestry) and the number of synonymous and nonsynonymous substitutions found in a non-overlapping window of a given genetic size. Ancestry was summarized using the results of an HMM run on a set of thinned input ancestry informative sites (see Methods). Minor parent ancestry is reduced in regions with a higher number of both synonymous and nonsynonymous substitutions between *X. birchmanni* and *X. malinche*. This may be driven by a correlation between coding substitutions and regions with a high number of linked coding basepairs (S7 Table).

| Population | nt change | Spearman’s correlation with minor parent ancestry | | | |
| --- | --- | --- | --- | --- | --- |
|  |  | **0.1 cM** | **0.25 cM** | **0.5 cM** | **1 cM** |
| Santa Cruz | non-synonymous | *ρ* = -0.10  p = 10^-30^ | *ρ* = -0.13  p = 10^-23^ | *ρ* = -0.16  p = 10^-20^ | *ρ* = -0.20  p = 10^-16^ |
|  | synonymous | *ρ* = -0.12  p = 10^-49^ | *ρ* = -0.16  p = 10^-36^ | *ρ* = -0.19  p = 10^-28^ | *ρ* = -0.25  p = 10^-24^ |
| Huextetitla | non-synonymous | *ρ* = -0.05  p = 10^-11^ | *ρ* = -0.08  p = 10^-10^ | *ρ* = -0.11  p = 10^-10^ | *ρ* = -0.14  p = 10^-9^ |
|  | synonymous | *ρ* = -0.08  p = 10^-21^ | *ρ* = -0.11  p = 10^-17^ | *ρ* = -0.14  p = 10^-15^ | *ρ* = -0.18  p = 10^-14^ |
