# Supplementary material for: Predictability and parallelism in the contemporary evolution of hybrid genomes
Source: PLoS Genet. 2022 Jan 27;18(1):e1009914. doi: 10.1371/journal.pgen.1009914 (PMC8794199; doi:10.1371/journal.pgen.1009914)
Supplement: S7 Table — Ancestry was summarized using the results of an HMM run on a set of thinned input ancestry informative sites (see Methods). (DOCX) [file pgen.1009914.s008.docx]

**S7 Table.** Relationship between minor parent ancestry (*X. birchmanni* ancestry), the number of coding basepairs, and the number of synonymous and nonsynonymous substitutions found in a window of a given genetic size. Ancestry was summarized using the results of an HMM run on a set of thinned input ancestry informative sites (see Methods).

| Population | nt change | Spearman’s partial correlation | | | |
| --- | --- | --- | --- | --- | --- |
|  |  | **0.1 cM** | **0.25 cM** | **0.5 cM** | **1 cM** |
| Santa Cruz | non-synonymous | *ρ* = 0.06  p = 10^-12^ | *ρ* = 0.10  p = 10^-15^ | *ρ* = 0.13  p = 10^-13^ | *ρ* = 0.17  p = 10^-12^ |
|  | synonymous | *ρ* = 0.04  p = 10^-7^ | *ρ* = 0.08  p = 10^-11^ | *ρ* = 0.12  p = 10^-11^ | *ρ* = 0.11  p = 10^-6^ |
|  | coding | *ρ* = -0.14  p = 10^-64^ | *ρ* = -0.22  p = 10^-66^ | *ρ* = -0.28  p = 10^-60^ | *ρ* = -0.33  p = 10^-44^ |
| Huextetitla | non-synonymous | *ρ* = 0.06  p = 10^-11^ | *ρ* = 0.09  p = 10^-12^ | *ρ* = 0.11  p = 10^-10^ | *ρ* = 0.15  p = 10^-9^ |
|  | synonymous | *ρ* = 0.04  p = 10^-05^ | *ρ* = 0.08  p = 10^-10^ | *ρ* = 0.11  p = 10^-10^ | *ρ* = 0.11  p = 10^-6^ |
|  | coding | *ρ* = -0.11  p = 10^-40^ | *ρ* = -0.19  p = 10^-49^ | *ρ* = -0.24  p = 10^-43^ | *ρ* = -0.30  p = 10^-35^ |
