# Supplementary material for: Predictability and parallelism in the contemporary evolution of hybrid genomes
Source: PLoS Genet. 2022 Jan 27;18(1):e1009914. doi: 10.1371/journal.pgen.1009914 (PMC8794199; doi:10.1371/journal.pgen.1009914)
Supplement: S8 Table — Ancestry was summarized using the results of an HMM run on a set of thinned input ancestry informative sites (see Methods). (DOCX) [file pgen.1009914.s009.docx]

**S8 Table.** Analysis of the correlation between minor parent ancestry and linked coding and conserved basepairs in 0.25 cM non-overlapping windows. Ancestry was summarized using the results of an HMM run on a set of thinned input ancestry informative sites (see Methods).

| Population | Spearman’s partial correlation with minor parent ancestry | | |
| --- | --- | --- | --- |
|  | **0.25 cM** | |  |
|  | **Coding** | **Conserved** |  |
| Santa Cruz | *ρ* =0.05  p = 10^-4^ | *ρ* = -0.20  p= 10^-55^ |  |
| Huextetitla | *ρ* =0.06  p= 10^-5^ | *ρ* = -0.17  p= 10^-40^ |  |
