# Supplementary material for: Predictability and parallelism in the contemporary evolution of hybrid genomes
Source: PLoS Genet. 2022 Jan 27;18(1):e1009914. doi: 10.1371/journal.pgen.1009914 (PMC8794199; doi:10.1371/journal.pgen.1009914)
Supplement: S9 Table — Here, windows in the lower or upper 25% of ancestry informative sites were dropped to exclude windows where we had especially low or high power to infer ancestry. (DOCX) [file pgen.1009914.s010.docx]

**S9 Table.** Analysis of the correlation between minor parent ancestry and linked conserved basepairs in non-overlapping windows in a filtered dataset. Here, windows in the lower or upper 25% of ancestry informative sites were dropped to exclude windows where we had especially low or high power to infer ancestry.

| Population | Spearman’s partial correlation with minor parent ancestry | | | | |
| --- | --- | --- | --- | --- | --- |
|  | **0.05 cM** | **0.1 cM** | | **0.25 cM** | |
| Santa Cruz | *ρ* = -0.1  p<10^-36^ | | *ρ* = -0.12  p<10^-28^ | | *ρ* = -0.17  p<10^-22^ |
| Huextetitla | *ρ* = -0.07  p<10^-18^ | | *ρ* = -0.09  p<10^-16^ | | *ρ* = -0.13  p<10^-14^ |
