# Supplementary material for: Predictability and parallelism in the contemporary evolution of hybrid genomes
Source: PLoS Genet. 2022 Jan 27;18(1):e1009914. doi: 10.1371/journal.pgen.1009914 (PMC8794199; doi:10.1371/journal.pgen.1009914)
Supplement: S10 Table — Santa Cruz and Huextetitla populations are X. birchmanni × X. cortezi hybrid populations that derive the majority of their genome from X. cortezi. Acuapa and Aguazarca are X. birchmanni × X. malinche hybrid populations that derive the majority of their genome from X. birchmanni; Tlatemaco is a X. birchmanni × X. malinche hybrid population that derives the majority of its genome from X. malinche. (DOCX) [file pgen.1009914.s011.docx]

**S10 Table**. Cross-population correlations in minor parent ancestry at a range of non-overlapping window sizes, without controlling for recombination rate and coding/conserved basepair covariates. Santa Cruz and Huextetitla populations are *X. birchmanni* × *X. cortezi* hybrid populations that derive the majority of their genome from *X. cortezi*. Acuapa and Aguazarca are *X. birchmanni* × *X. malinche* hybrid populations that derive the majority of their genome from *X. birchmanni*; Tlatemaco is a *X. birchmanni* × *X. malinche* hybrid population that derives the majority of its genome from *X. malinche*.

| Population | Comparison Population | Spearman’s correlation with minor parent ancestry | | |
| --- | --- | --- | --- | --- |
|  |  | **50 kb** | **100 kb** | **250 kb** |
| Santa Cruz | Huextetitla | *ρ* = 0.75  p < 10^-325^ | *ρ* = 0.78  p < 10^-325^ | *ρ* = 0.82  p < 10^-325^ |
|  | Tlatemaco | *ρ* = 0.01  p = 0.15 | *ρ* = 0.02  p = 0.19 | *ρ* = 0.03  p = 0.15 |
|  | Acuapa | *ρ* = 0.23  p = 10^-159^ | *ρ* = 0.25  p = 10^-93^ | *ρ* = 0.28  p = 10^-49^ |
|  | Aguazarca | *ρ* = 0.15  p = 10^-74^ | *ρ* = 0.17  p = 10^-42^ | *ρ* = 0.19  p = 10^-23^ |
| Huextetitla | Tlatemaco | *ρ* = 0.02  p = 0.06 | *ρ* = 0.02  p = 0.14 | *ρ* = 0.03  p = 0.12 |
|  | Acuapa | *ρ* = 0.23  p = 10^-163^ | *ρ* = 0.25  p = 10^-97^ | *ρ* = 0.29  p = 10^-55^ |
|  | Aguazarca | *ρ* = 0.15  p = 10^-75^ | *ρ* = 0.17  p = 10^-44^ | *ρ* = 0.19  p= 10^-24^ |
| Tlatemaco | Acuapa | *ρ* = -0.08  p = 10^-19^ | *ρ* = -0.07  p = 10^-9^ | *ρ* = -0.06  p = 10^-3^ |
|  | Aguazarca | *ρ* = -0.07  p = 10^-16^ | *ρ* = -0.07  p = 10^-8^ | *ρ* = -0.06  p = 0.002 |
| Acuapa | Aguazarca | *ρ* = 0.36  p < 10^-325^ | *ρ* = 0.36  p = 10^-208^ | *ρ* = 0.38  p = 10^-92^ |
