# Supplementary material for: Predictability and parallelism in the contemporary evolution of hybrid genomes
Source: PLoS Genet. 2022 Jan 27;18(1):e1009914. doi: 10.1371/journal.pgen.1009914 (PMC8794199; doi:10.1371/journal.pgen.1009914)
Supplement: S12 Table — For each population and window size comparison, Spearman’s ρ from cross-population ancestry correlations after accounting for other features is given to the left of each cell. The estimated ρ from the same partial correlation analysis of other features (recombination rate, number of coding basepairs, and number of conserved basepairs) are given one the right. (DOCX) [file pgen.1009914.s013.docx]

**S12 Table.** Cross-population correlations in minor parent ancestry at a range of non-overlapping window sizes, including recombination rate, coding, and conserved basepair covariates. For each population and window size comparison, Spearman’s *ρ* from cross-population ancestry correlations after accounting for other features is given to the left of each cell. The estimated *ρ* from the same partial correlation analysis of other features (recombination rate, number of coding basepairs, and number of conserved basepairs) are given one the right.

| Population | Cross Population | Spearman’s partial correlation with minor parent ancestry | | | | | | |
| --- | --- | --- | --- | --- | --- | --- | --- | --- |
|  |  | **50 kb** | | **100 kb** | | **250 kb** | |  |
| Santa cruz | Huextetitla | *ρ* = 0.71  p<10^-325^ | rec *ρ* = 0.20  coding *ρ* = 0.04  conserved *ρ* = -0.03 | *ρ* = 0.73  p<10^-325^ | rec *ρ* = 0.20  coding *ρ* = 0.04  conserved *ρ* = -0.03 | *ρ* = 0.77  p<10^-325^ | rec *ρ* = 0.19  coding *ρ* = 0.04  conserved *ρ* = -0.03 |  |
|  | Tlatemaco | *ρ* = 0.00  p =0.88 | rec *ρ* = 0.39  coding *ρ* = 0.10  conserved *ρ* = -0.08 | *ρ* = 0.00  p =0.85 | rec *ρ* = 0.43  coding *ρ* = 0.11  conserved *ρ* = -0.09 | *ρ* = 0.01  p =0.63 | rec *ρ* = 0.50  coding *ρ* = 0.11  conserved *ρ* = -0.10 |  |
|  | Acuapa | *ρ* = 0.15  p =10^-64^ | rec *ρ* = 0.36  coding *ρ* = 0.10  conserved *ρ* = -0.08 | *ρ* = 0.15  p =10^-33^ | rec *ρ* = 0.39  coding *ρ* = 0.11  conserved *ρ* = -0.09 | *ρ* = 0.16  p =10^-16^ | rec *ρ* = 0.46  coding *ρ* = 0.11  conserved *ρ* = -0.10 |  |
|  | Aguazarca | *ρ* = 0.10  p =10^-30^ | rec *ρ* = 0.38  coding *ρ* = 0.10  conserved *ρ* = -0.08 | *ρ* = 0.09  p =10^-15^ | rec *ρ* = 0.42  coding *ρ* = 0.10  conserved *ρ* = -0.09 | *ρ* = 0.10  p =10^-7^ | rec *ρ* = 0.49  coding *ρ* = 0.11  conserved *ρ* = -0.09 |  |
| Huextetitla | Santa Cruz | *ρ* = 0.71  p<10^-325^ | rec *ρ* = 0.11  coding *ρ* = 0.05  conserved *ρ* = -0.04 | *ρ* = 0.73  p<10^-325^ | rec *ρ* = 0.13  coding *ρ* = 0.05  conserved *ρ* = -0.05 | *ρ* = 0.77  p <10^-325^ | rec *ρ* = 0.16  coding *ρ* = 0.05  conserved *ρ* = -0.05 |  |
|  | Tlatemaco | *ρ* = 0.01  p =0.48 | rec *ρ* = 0.36  coding *ρ* = 0.11  conserved *ρ* = -0.09 | *ρ* = 0.01  p =0.64 | rec *ρ* = 0.41  coding *ρ* = 0.11  conserved *ρ* = -0.10 | *ρ* = 0.01  p =0.51 | rec *ρ* = 0.49  coding *ρ* = 0.12  conserved *ρ* = -0.10 |  |
|  | Acuapa | *ρ* = 0.16  p =10^-73^ | rec *ρ* = 0.32  coding *ρ* = 0.11  conserved *ρ* = -0.09 | *ρ* = 0.16  p =10^-39^ | rec *ρ* = 0.37  coding *ρ* = 0.12  conserved *ρ* = -0.10 | *ρ* = 0.18  p =10^-21^ | rec *ρ* = 0.45  coding *ρ* = 0.12  conserved *ρ* = -0.10 |  |
|  | Aguazarca | *ρ* = 0.10  p =10^-33^ | rec *ρ* = 0.35  coding *ρ* = 0.10  conserved *ρ* = -0.08 | *ρ* = 0.10  p =10^-17^ | rec *ρ* = 0.39  coding *ρ* = 0.11  conserved *ρ* = -0.09 | *ρ* = 0.10  p=10^-7^ | rec *ρ* = 0.48  coding *ρ* = 0.11  conserved *ρ* = -0.10 |  |
