# Supplementary material for: Predictability and parallelism in the contemporary evolution of hybrid genomes
Source: PLoS Genet. 2022 Jan 27;18(1):e1009914. doi: 10.1371/journal.pgen.1009914 (PMC8794199; doi:10.1371/journal.pgen.1009914)
Supplement: S13 Table — (DOCX) [file pgen.1009914.s014.docx]

**S13 Table.** Cross-population correlations in minor parent ancestry at a range of genetic non-overlapping window sizes.

| Population | Cross Population | Spearman’s correlation with minor parent ancestry | | | |
| --- | --- | --- | --- | --- | --- |
|  |  | **0.1 cM** | **0.25 cM** | **0.5 cM** | **1 cM** |
| Santa Cruz | Huextetitla | *ρ* = 0.87  p < 10^-325^ | *ρ* = 0.89  p < 10^-325^ | *ρ* = 0.91  p < 10^-325^ | *ρ* = 0.93  p < 10^-325^ |
|  | Tlatemaco | *ρ* = -0.03  p = 10^-5^ | *ρ* = -0.03  p = 0.03 | *ρ* = -0.01  p = 0.49 | *ρ* = 0.00  p = 0.94 |
|  | Acuapa | *ρ* = 0.26  p = 10^-205^ | *ρ* = 0.28  p = 10^-106^ | *ρ* = 0.30  p = 10^-66^ | *ρ* = 0.33  p = 10^-43^ |
|  | Aguazarca | *ρ* = 0.19  p = 10^-105^ | *ρ* = 0.20  p = 10^-57^ | *ρ* = 0.23  p = 10^-37^ | *ρ* = 0.26  p = 10^-27^ |
| Huextetitla | Santa Cruz | *ρ* = 0.87  p < 10^-325^ | *ρ* = 0.89  p < 10^-325^ | *ρ* = 0.91  p < 10^-325^ | *ρ* = 0.93  p < 10^-325^ |
|  | Tlatemaco | *ρ* = -0.03  p = 0.003 | *ρ* = -0.01  p = 0.30 | *ρ* = 0.1  p = 0.69 | *ρ* = 0.02  p = 0.54 |
|  | Acuapa | *ρ* = 0.22  p = 10^-152^ | *ρ* = 0.24  p = 10^-81^ | *ρ* = 0.27  p = 10^-52^ | *ρ* = 0.30  p = 10^-36^ |
|  | Aguazarca | *ρ* = 0.17  p = 10^-84^ | *ρ* = 0.19  p = 10^-49^ | *ρ* = 0.22  p= 10^-35^ | *ρ* = 0.25  p= 10^-25^ |
