# Supplementary figures and images for: Predictability and parallelism in the contemporary evolution of hybrid genomes
Source: PLoS Genet. 2022 Jan 27;18(1):e1009914. doi: 10.1371/journal.pgen.1009914 (PMC8794199; doi:10.1371/journal.pgen.1009914)

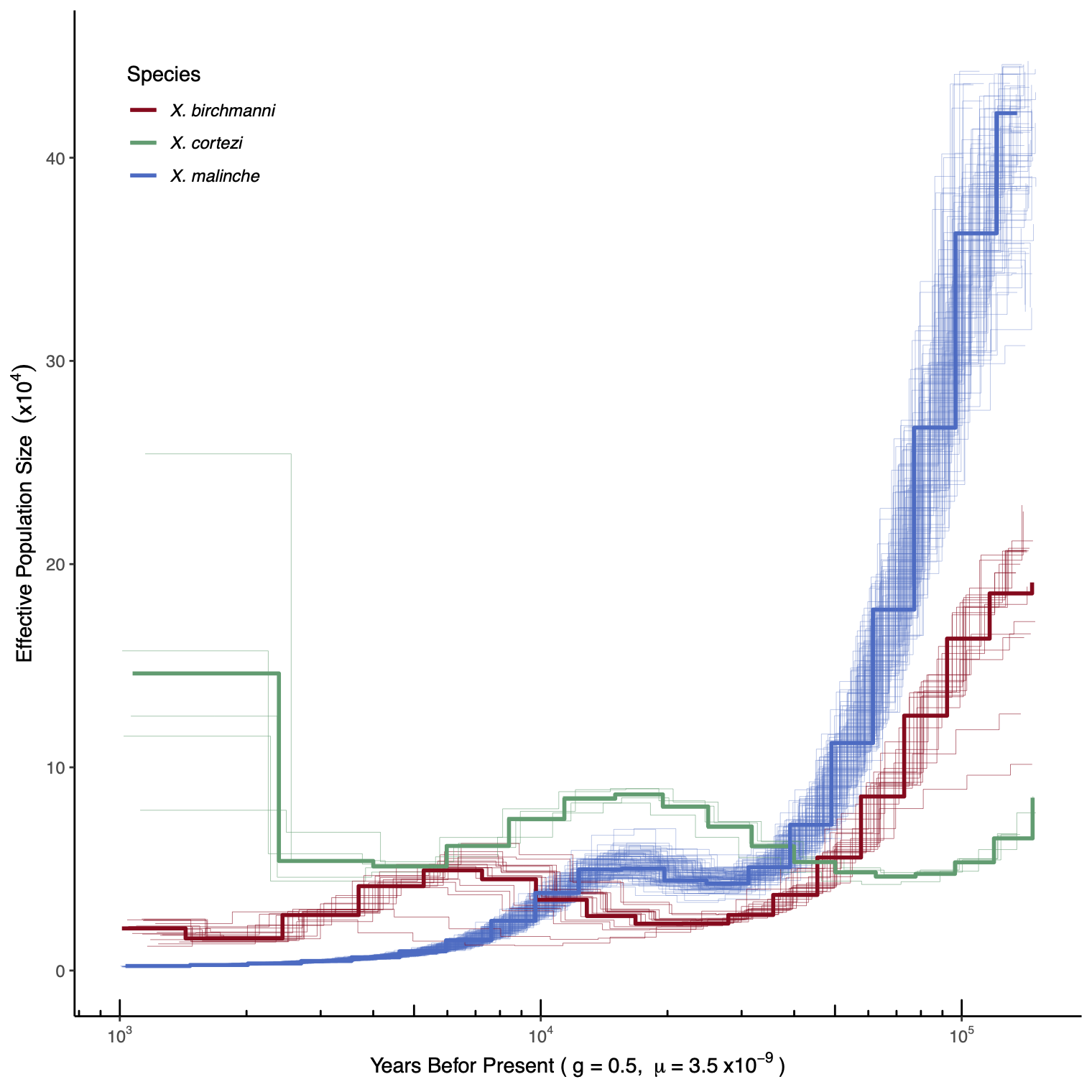

Supplement: S1 Fig — For visualization the single X. malinche sample was bootstrapped 100 times by resampling with replacement from the genome split into 500 kb segments. Analysis was conducted similarly to [5] with the time segmentation parameter set to 4+25*2+4+6, a ρ/θ ratio of 2, generation time of two generations per year, and mutation rate of 3.5 × 10^−9^. (TIFF) [file pgen.1009914.s021.tiff]

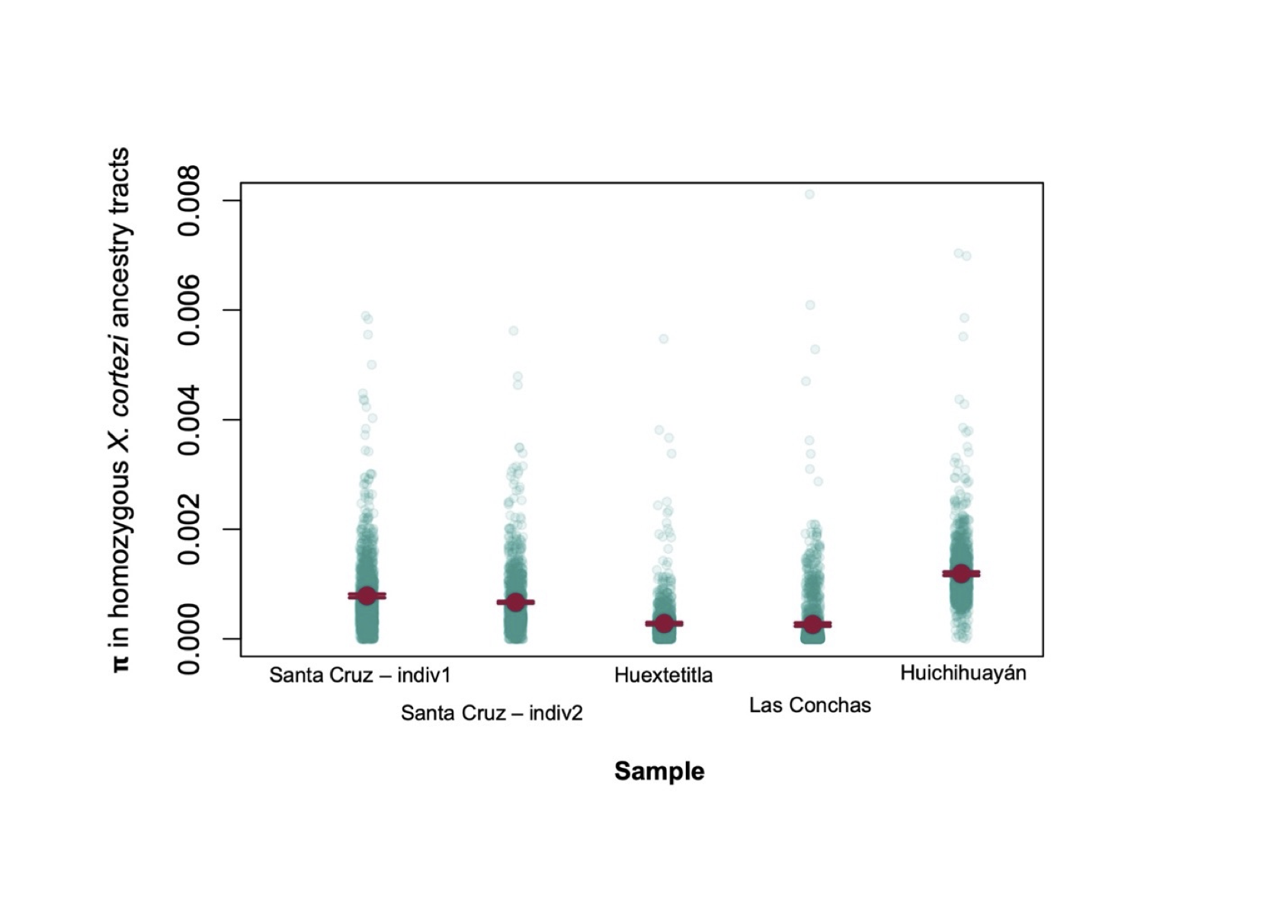

Supplement: S2 Fig — (TIFF) [file pgen.1009914.s022.tiff]

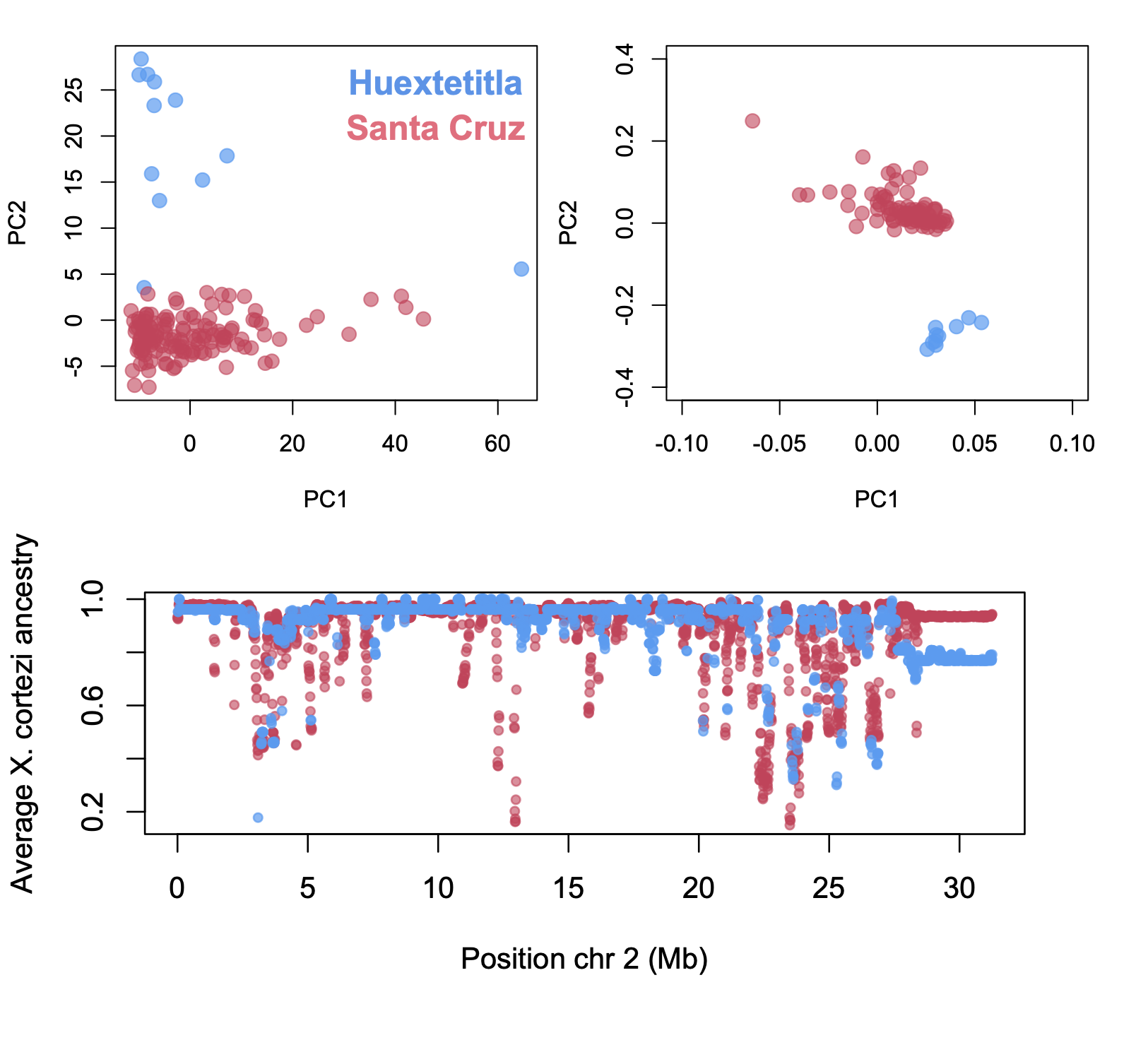

Supplement: S3 Fig — (Top) (Left) PCA analysis of the locations of observed ancestry transitions in individuals from the Santa Cruz and Huextetitla hybrid populations. (Right) PCA analysis of pseudohaploid SNP calls derived from low-coverage sequence data of individuals from the Santa Cruz and Huextetitla populations (see Text B in S1 File). Separation along PC2 suggests that the Santa Cruz and Huextetitla hybrid populations have been somewhat independent in their recent demographic histories. (Bottom) Example of heterogeneity in ancestry along chromosome 2 in Huextetitla (blue) and Santa Cruz (pink) X. birchmanni × X. cortezi hybrid populations, averaged in 10 kb windows). Both populations have regions that are fixed or nearly fixed for both X. cortezi and X. birchmanni ancestry. Note the strong correlations in local ancestry between the two populations (ρ = 0.65, p<10−100). (TIFF) [file pgen.1009914.s023.tiff]

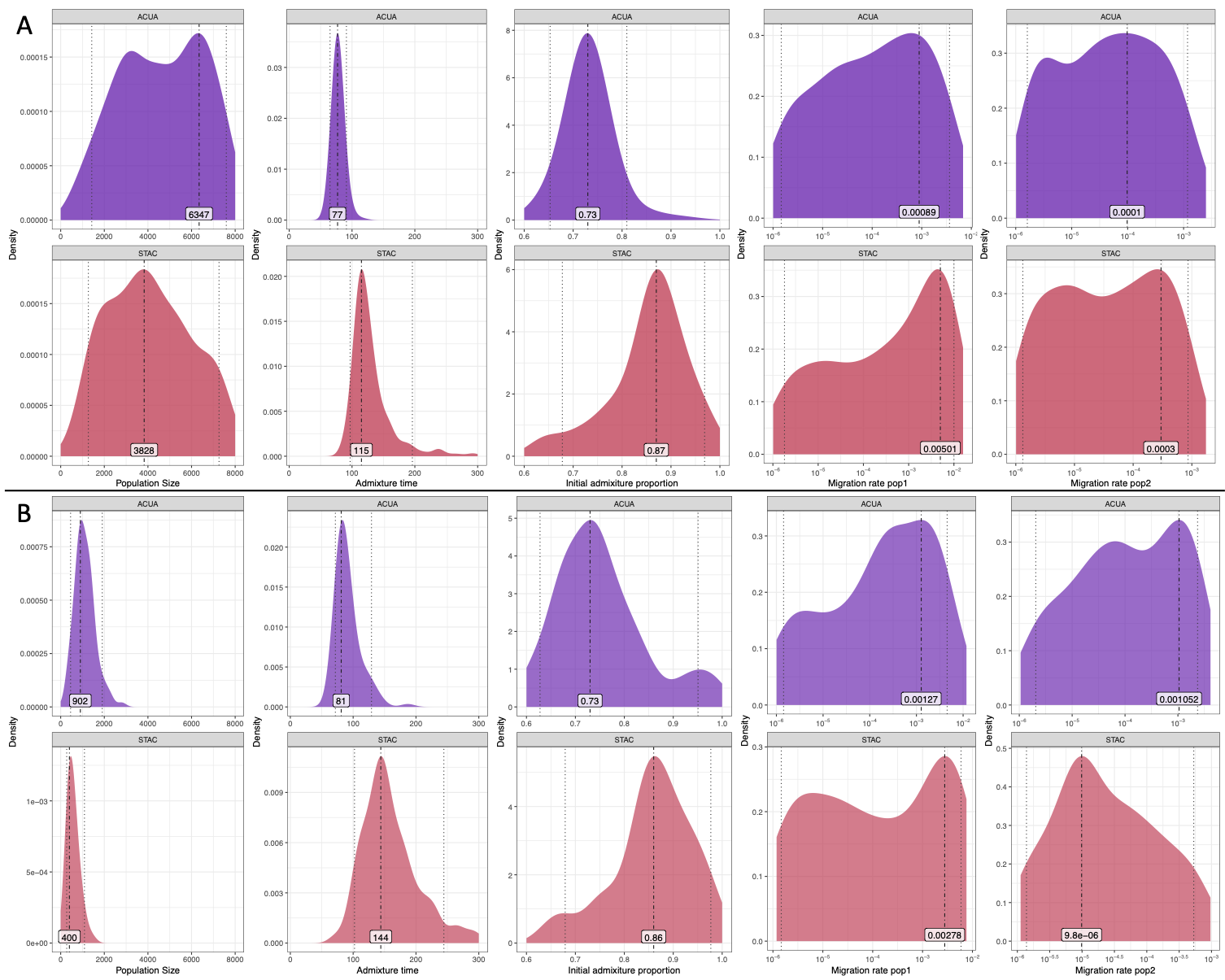

Supplement: S4 Fig — Posterior distributions from Approximate Bayesian Computation (ABC) simulations used to infer the demographic history for a X. birchmanni × X. cortezi hybrid population Santa Cruz (STAC) (red) and a X. birchmanni × X. malinche hybrid population Acuapa (ACUA) (pruple). Dot-dashed lines and listed values correspond to the maximum a posteriori or MAP estimate for each distribution. Dotted lines are the 95% quantile range. See Text C in S1 File for complete details of SLiM simulations and rejection sampling approach. Posterior distributions shown here are derived from uniform (or log-uniform) prior distributions of: initial population size, time since admixture (in generations), initial admixture proportion, and migration rate from each parent species. We accepted simulations based on two sets of summary statistics. A. Our primary analysis included summary statistics for the median length of minor parent ancestry tracts, average hybrid index, and the coefficient of variation for chromosome-wide ancestry across sampled individuals (ACUA N = 500, STAC N = 500). B. In a second analysis we included summary statistics for the median length of minor parent ancestry tracts, average hybrid index, and the coefficient of variation for local ancestry along the chromosome in 250 kb windows. We accepted very few simulations using the second approach (ACUA N = 98, STAC N = 90). We show the accepted simulations here to emphasize that the posterior distributions for most parameters are similar using the two approaches but rely on the inferences made in A for almost all analyses. See Text C in S1 File for additional information. (TIFF) [file pgen.1009914.s024.tiff]

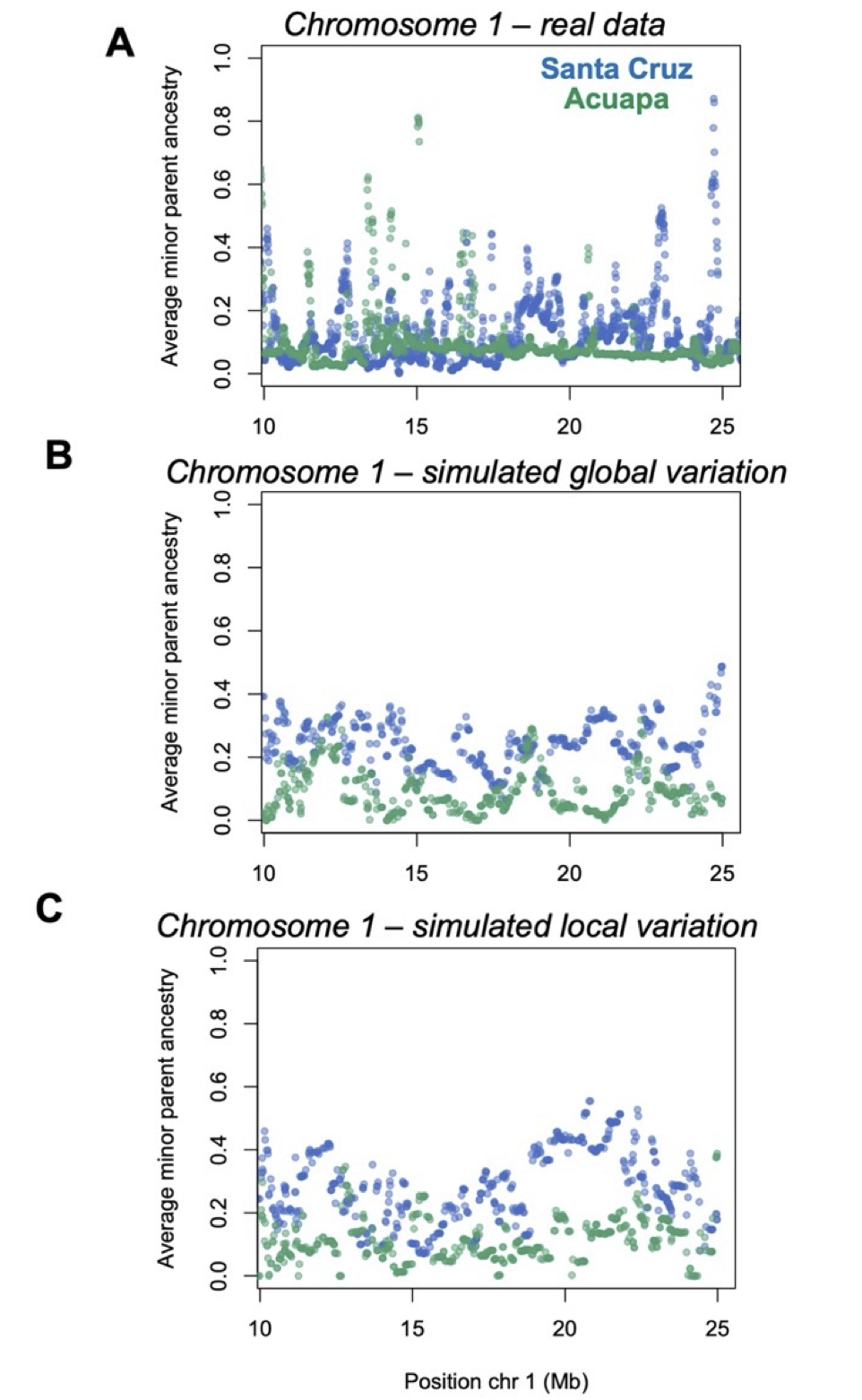

Supplement: S5 Fig — A. Heterogeneity in minor parent ancestry in the real data for a section of chromosome 1 in the Santa Cruz and Acuapa populations. Points show the average minor parent ancestry in 10 kb windows. B. Results of one replicate simulation of local ancestry on chromosome 1 for Santa Cruz and Acuapa based on randomly drawn set of demographic parameters from the posterior distribution of ABC simulations that used global variation in admixture proportion as a summary statistic (see Text C in S1 File & S4 Fig for details). C. Results of one replicate simulation of local ancestry on chromosome 1 for Santa Cruz and Acuapa based on randomly drawn set of demographic parameters from the posterior distribution of ABC simulations that used local variation in admixture proportion (summarized in 250 kb windows) as a summary statistic. Points show the average minor parent ancestry in 10 kb windows. While these simulations incorporated inferred demographic history for each population they did not implement selection. This results in lower heterogenetity in local ancestry compared to the real data, even when a summary statistic of local variation in admixture proportion was used to accept or reject simulations (see Text C in S1 File & S4 Fig). (TIFF) [file pgen.1009914.s025.tiff]

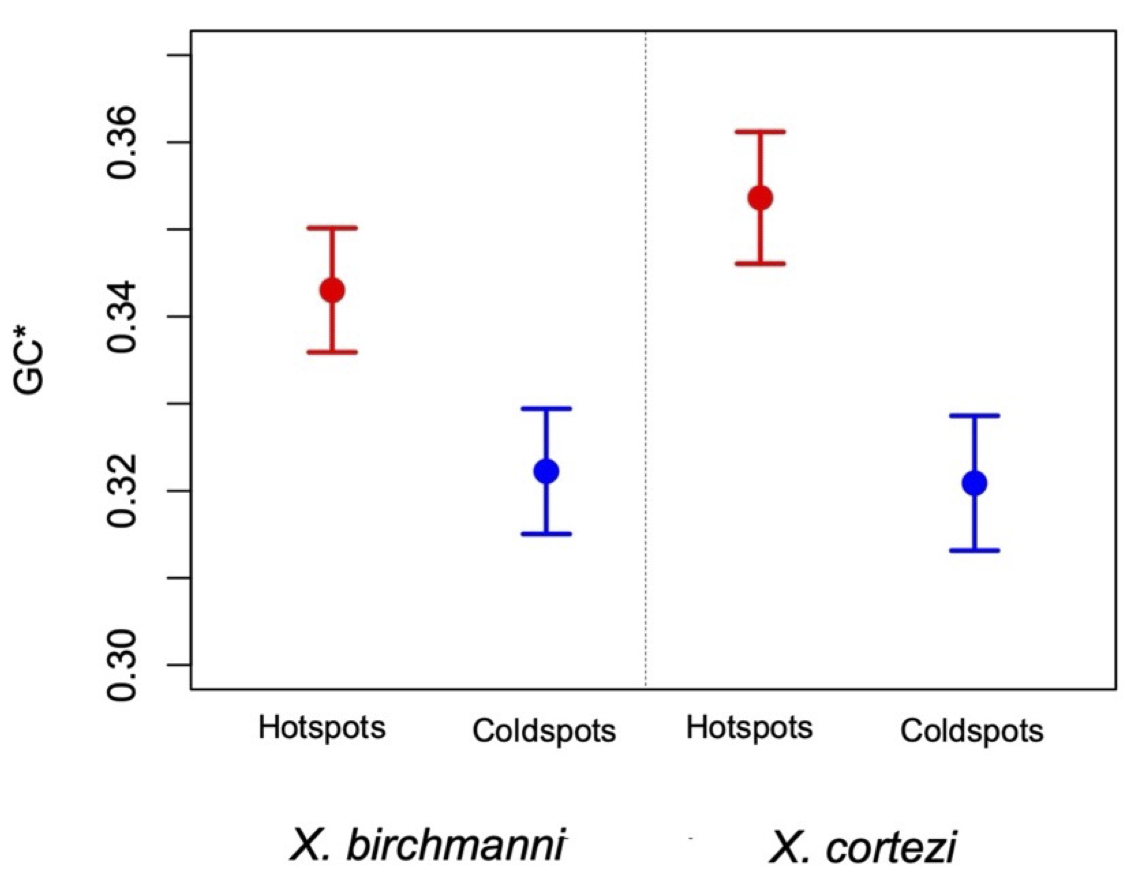

Supplement: S6 Fig — These patterns suggest an excess of GC-biased gene conversion in hotspots identified in X. birchmanni in both species, providing further evidence that the fine scale recombination maps are shared between species. (TIFF) [file pgen.1009914.s026.tiff]

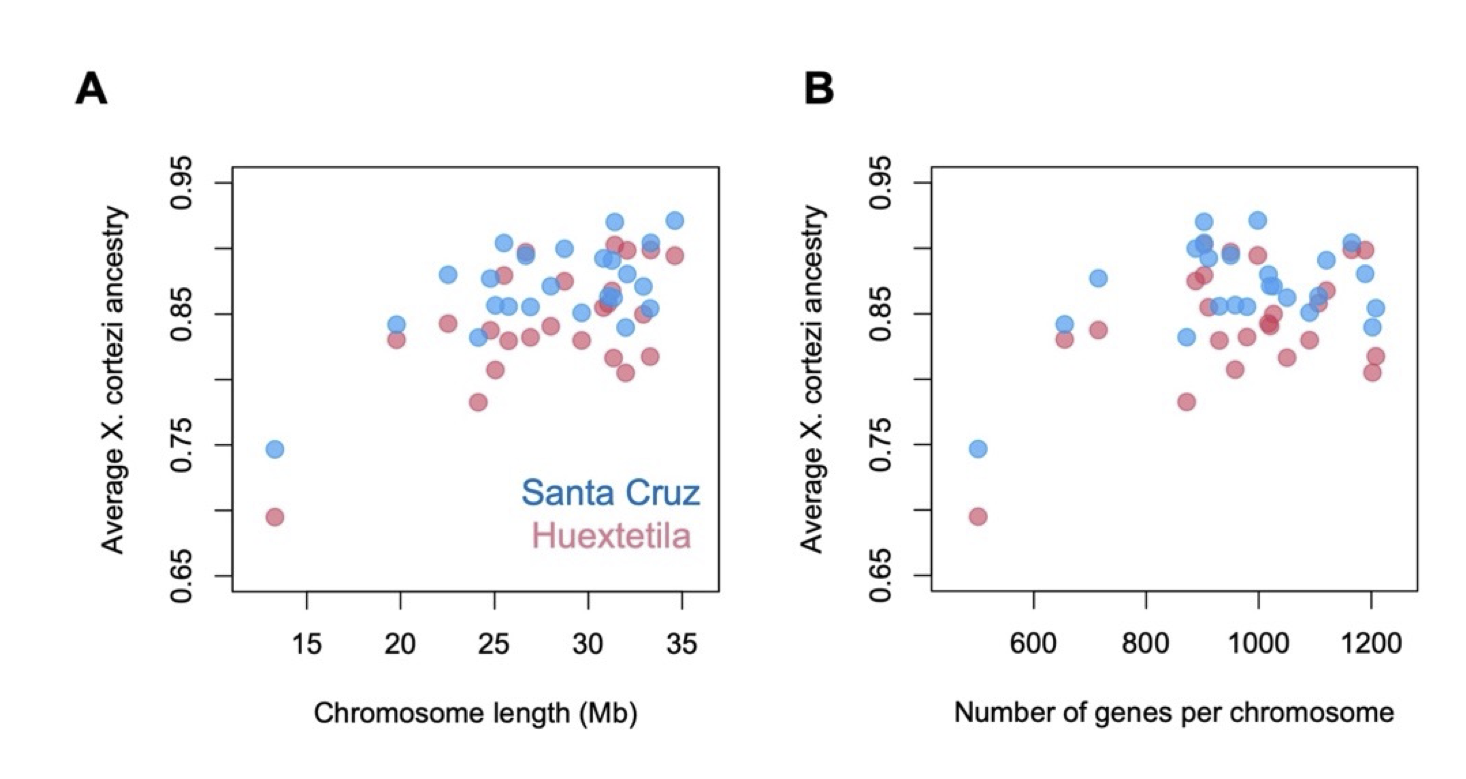

Supplement: S7 Fig — A. In both Santa Cruz and Huextetitla this heterogeneity is modestly correlated with chromosome length, suggesting that it may be driven by higher effective recombination rates on shorter chromosomes (ρSanta Cruz = 0.43, p Santa Cruz = 0.036; ρHuextetitla = 0.39, pHuextetitla = 0.058). B. The correlation between number of genes per chromosome and chromosome-level ancestry is substantially weaker (ρSanta Cruz = 0.12, pSanta Cruz = 0.58; ρHuextetitla = 0.03, pHuextetitla = 0.90). (TIFF) [file pgen.1009914.s027.tiff]

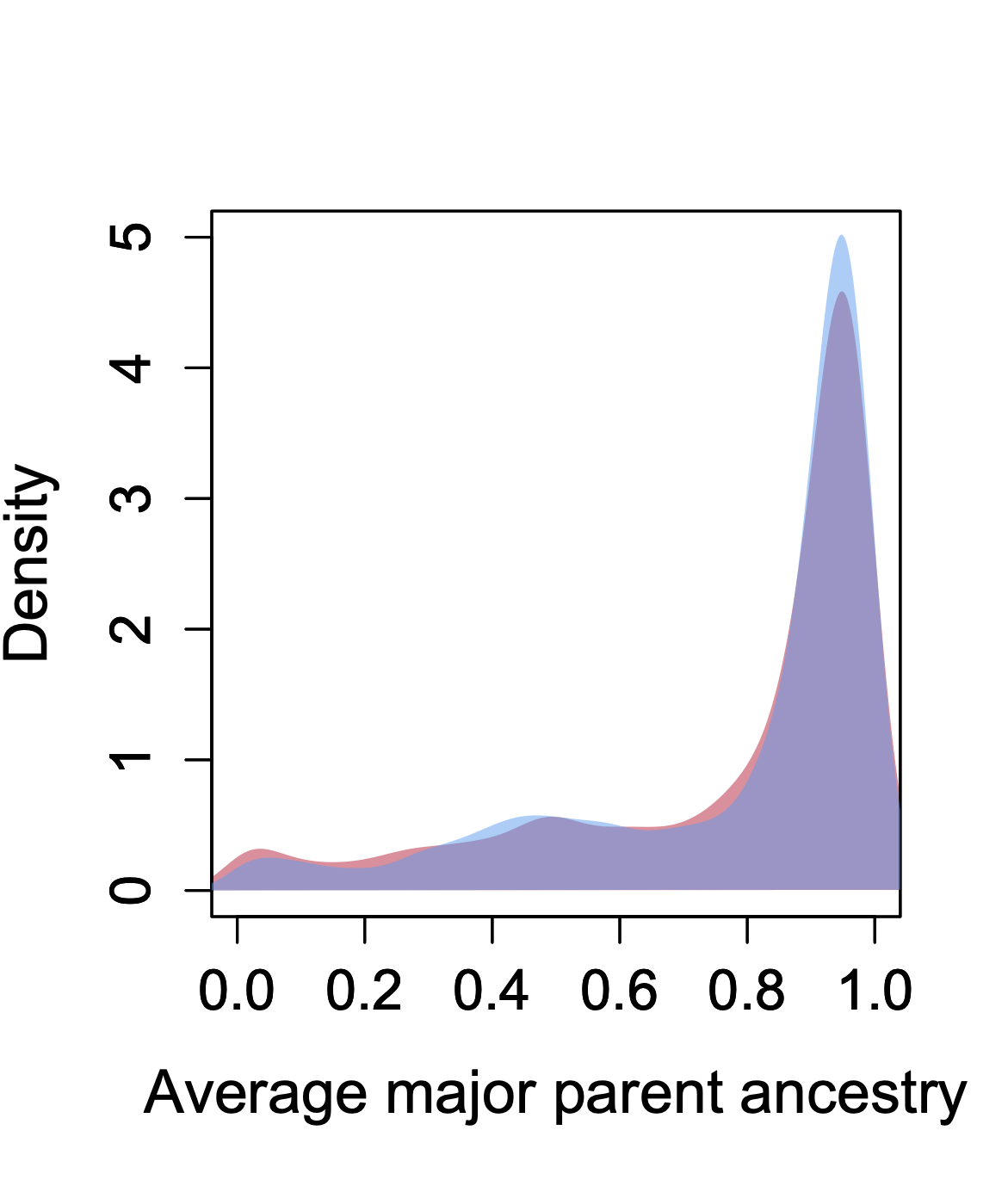

Supplement: S8 Fig — For each 0.1 cM window with a high number of nonsynonymous substitutions, we identified a 0.1 cM window with no nonsynonymous substitutions but an overall coding substitution rate (i.e. of synonymous substitutions) within 80–120% of that observed in the focal window. We see no significant differences in the minor parent ancestry distributions of the focal (pink) and matched (blue) windows. (TIFF) [file pgen.1009914.s028.tiff]

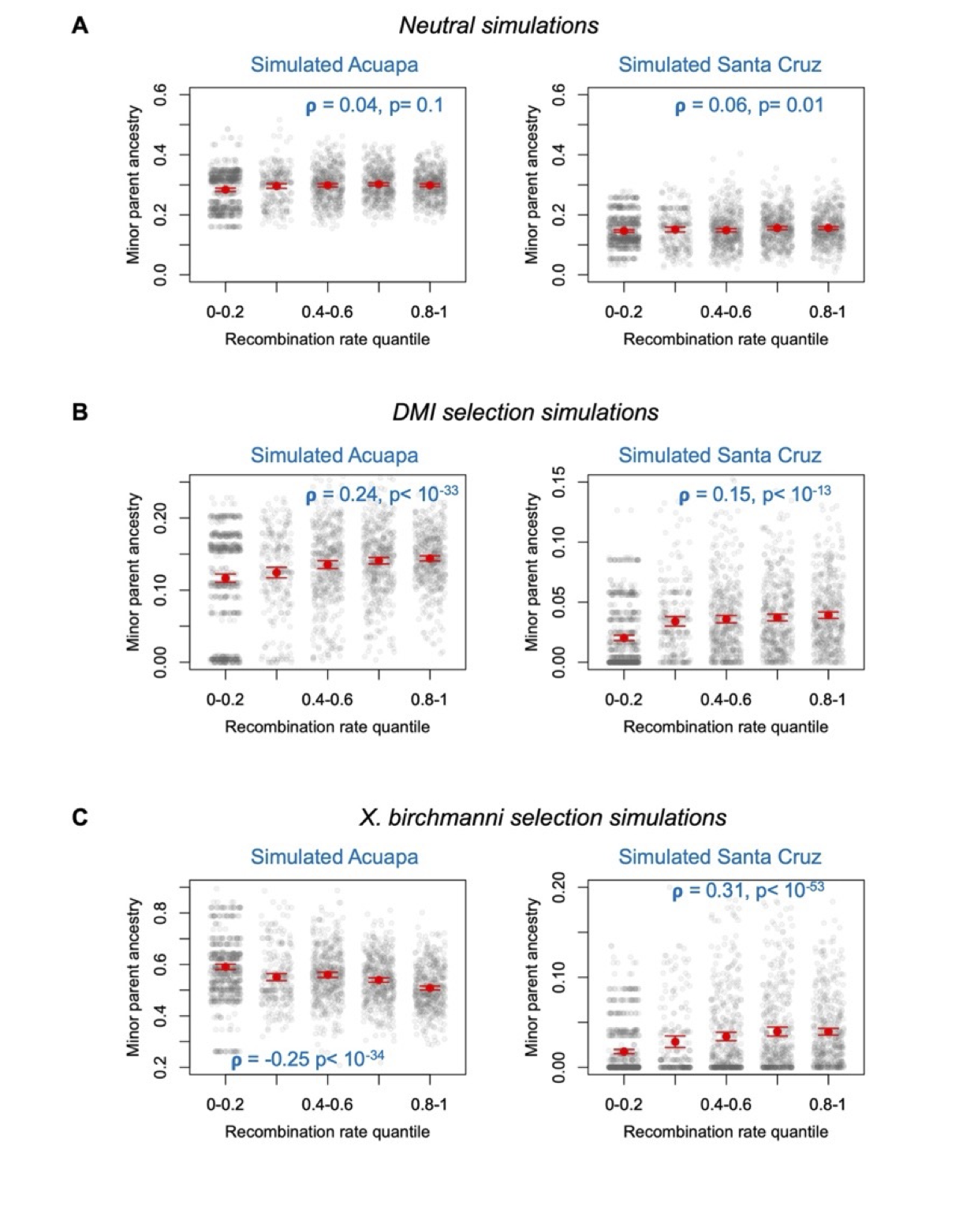

Supplement: S9 Fig — A. In the absence of selection there is no expected relationship between recombination rate and ancestry, and indeed this is what is observed in simulated Santa Cruz and Acuapa populations (single simulation example shown here). Gray points show minor parent ancestry in 250 kb windows, red points and whiskers show the mean and two standard errors of the mean. Inset shows correlation coefficient and p-value for the representative simulation. B. In the presence of selection against hybrid incompatibilities, selection drives a positive correlation between minor parent ancestry and recombination rate, regardless of the identity of the major parent species. Shown here are single representative simulations modeling the demographic history of the Santa Cruz and Acuapa populations with incompatibility selection implemented at 20 random pairs of sites throughout the genome. C. In the presence of selection against one parent species or the other, we expect to see conflicting directions in the correlation between minor parent ancestry and recombination rate depending on the admixture proportion of the hybrid population. In this set of simulations, a subset of sites derived from the X. birchmanni parent were globally disadvantageous, driving different patterns in the majority X. birchmanni (Acuapa) and minority X. birchmanni (Santa Cruz) hybrid populations. Simulations are described in detail in Text F in S1 File. (TIFF) [file pgen.1009914.s029.tiff]

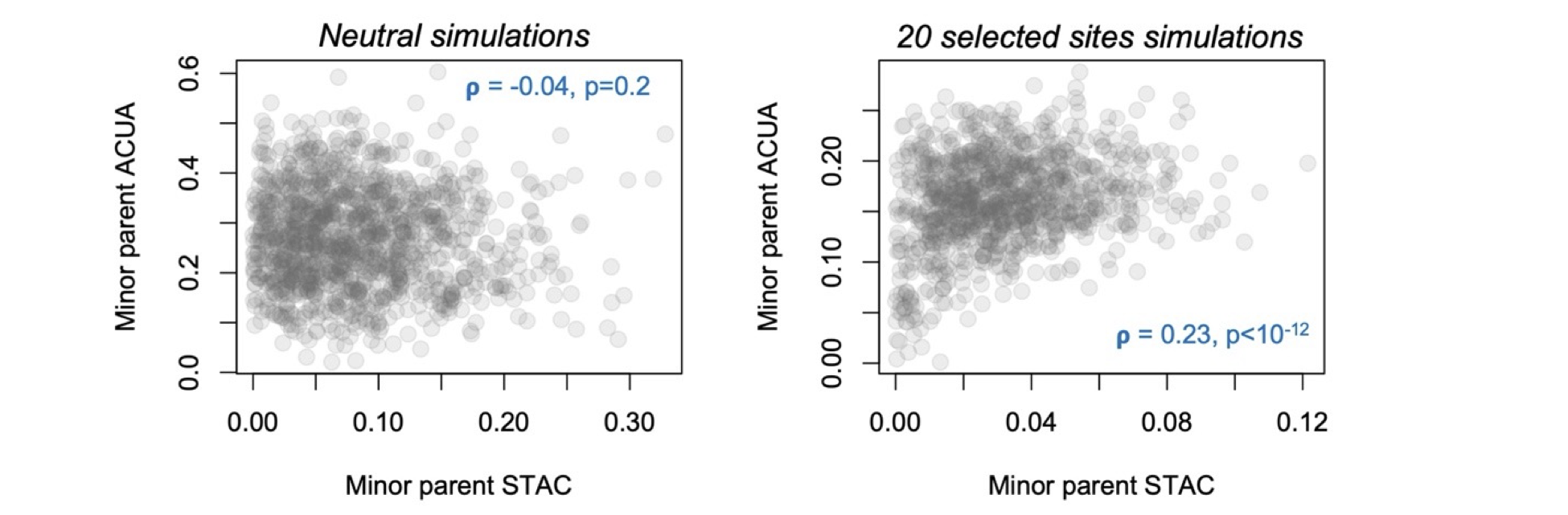

Supplement: S10 Fig — A. Example simulation of local ancestry in Santa Cruz and Acuapa populations modeling inferred demographic history but no selection. Inset shows correlation coefficient and p-value for the pair of simulations. B. Example simulation of local ancestry in Santa Cruz and Acuapa populations modeling inferred demographic history and 20 randomly placed shared sites under selection in the two populations. Blue text shows correlation coefficient and p-value for the pair of simulations. (TIFF) [file pgen.1009914.s030.tiff]

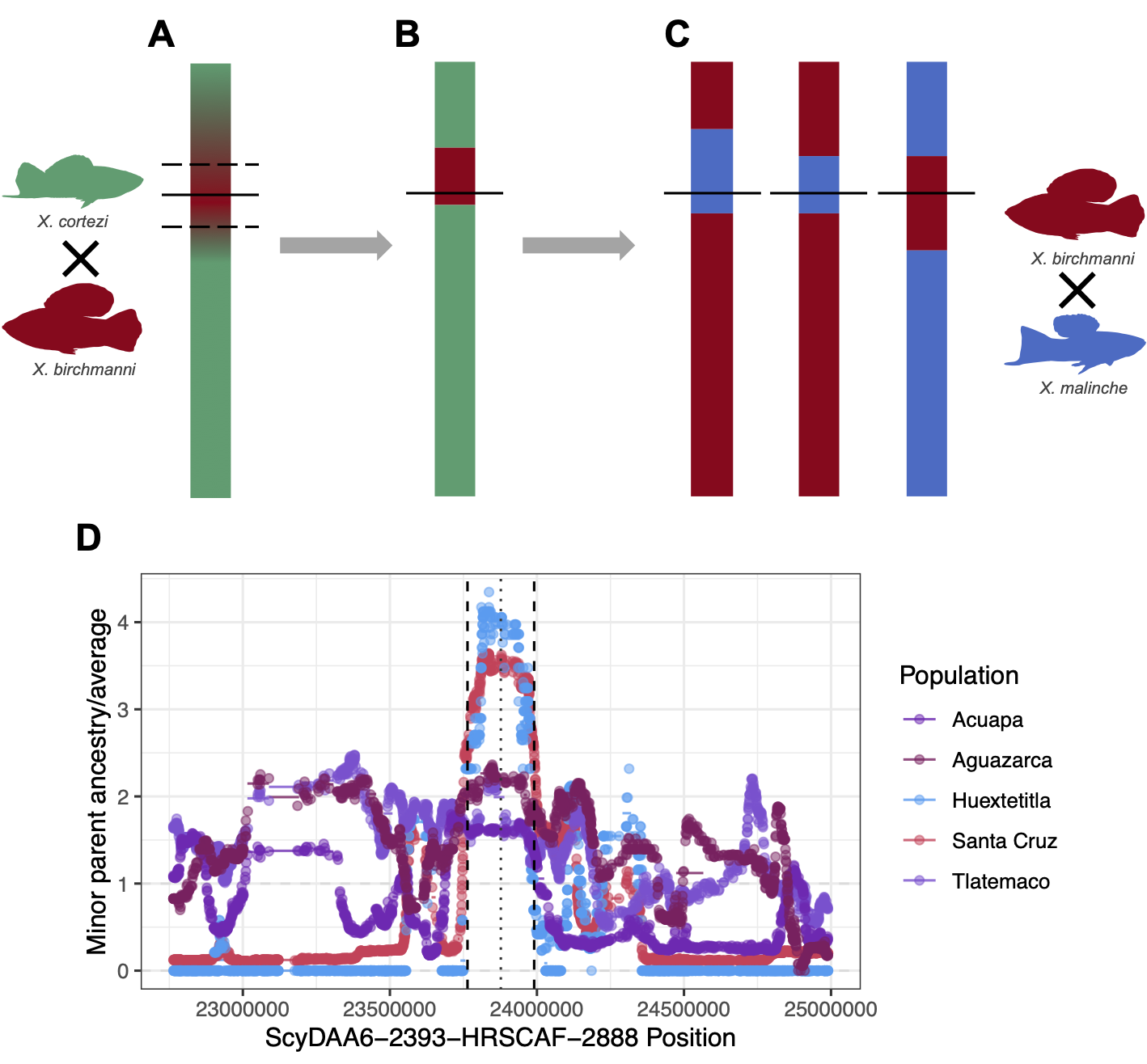

Supplement: S11 Fig — We employed a stepwise approach to identify deserts and islands of minor parent ancestry and determine if they were shared across populations. Shown here is a hypothetical workflow for identifying a shared minor parent ancestry island. A. We started with identifying AIMs where average minor parent ancestry at that site exceeded the 97.5% quantile of minor parent ancestry genome-wide. From that focal site we expanded outward in the 5’ and 3’ directions to identify where minor parent ancestry falls below the 95% tail of the genome-wide distribution. This set the boundary of the focal minor parent island region. We then determined the midpoint of each region and identified the 0.05 cM window that contains the midpoint. B. We checked that the focal population’s minor parent ancestry is greater than the 90% quantile of minor parent ancestry genome-wide when averaged across this 0.05 cM window. We then asked if this region is a shared minor parent ancestry outlier in other populations. C. Specifically, we evaluated minor parent ancestry in the midpoint 0.05 cM window in other hybrid populations. If minor parent ancestry in these populations is exceeded the 90% quantile of that population’s genome wide ancestry distribution we classified that region as a shared minor parent island. D. Example of a minor parent island detected with this work flow. Dashed lines are the identified boundaries of the island and dotted line is the midpoint. Colored dots correspond to the minor parent ancestry at a given ancestry informative site divided by the genome wide average for that population. Colored lines indicate the minor parent ancestry for the focal 0.05 cM window divided by the genome wide average for the population. (TIFF) [file pgen.1009914.s031.tiff]

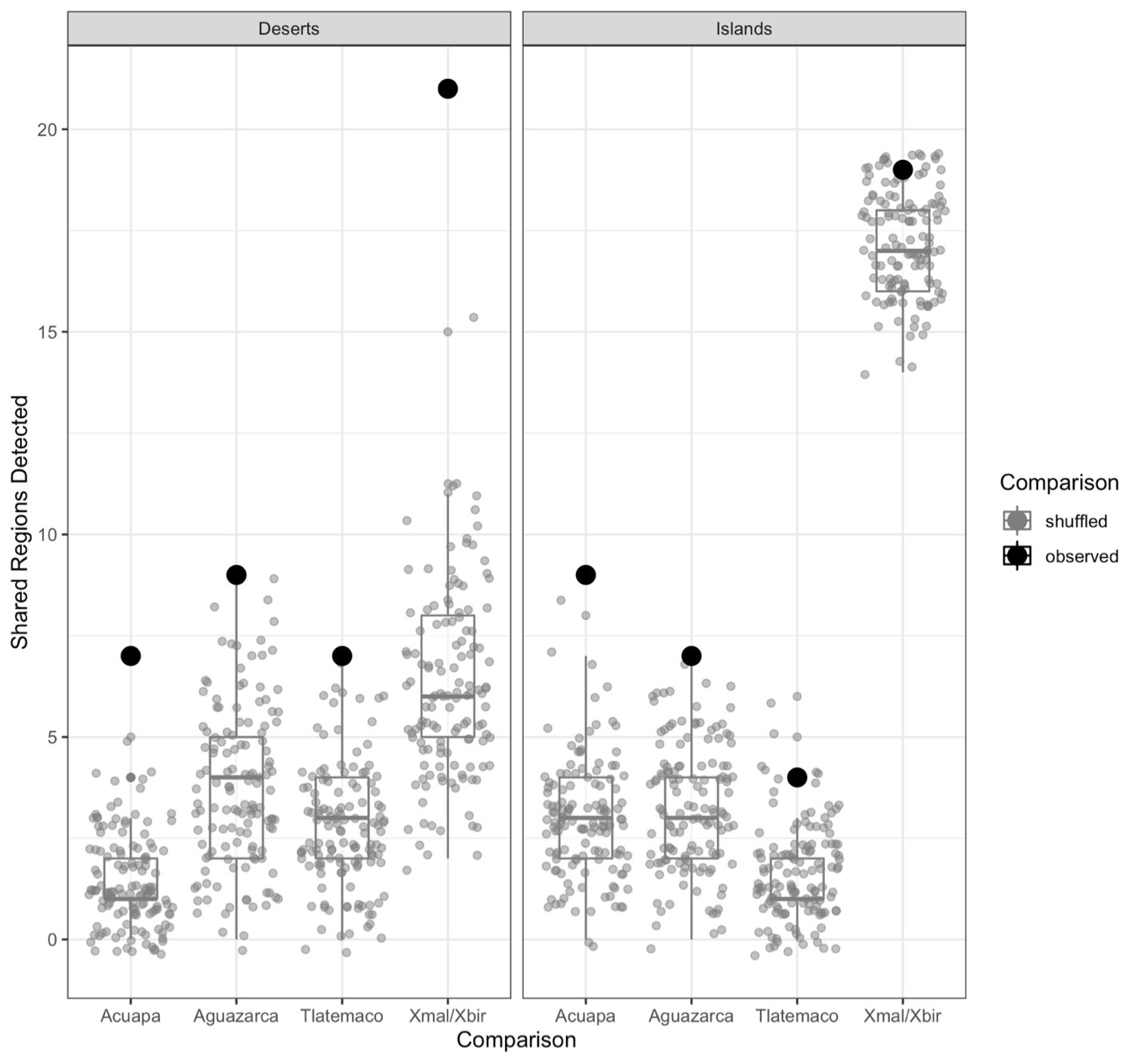

Supplement: S12 Fig — By contrast, minor parent islands are less enriched compared to null datasets when using this approach. Results shown here indicate the number of shared minor parent deserts (or islands) between the Santa Cruz X. birchmanni × X. cortezi hybrid population and each X. birchmanni × X. malinche hybrid population (Acuapa, Aguazarca, and Tlatemaco). Large black circles show the observed number of shared minor parent deserts or islands. Gray points and boxplots show the expectations from 130 shuffled datasets tiling the genome (see Text H in S1 File). The column labeled Xmal/Xbir shows the number of shared deserts or islands between the Santa Cruz X. birchmanni × X. cortezi hybrid population and any single X. birchmanni × X. malinche population. (TIFF) [file pgen.1009914.s032.tiff]

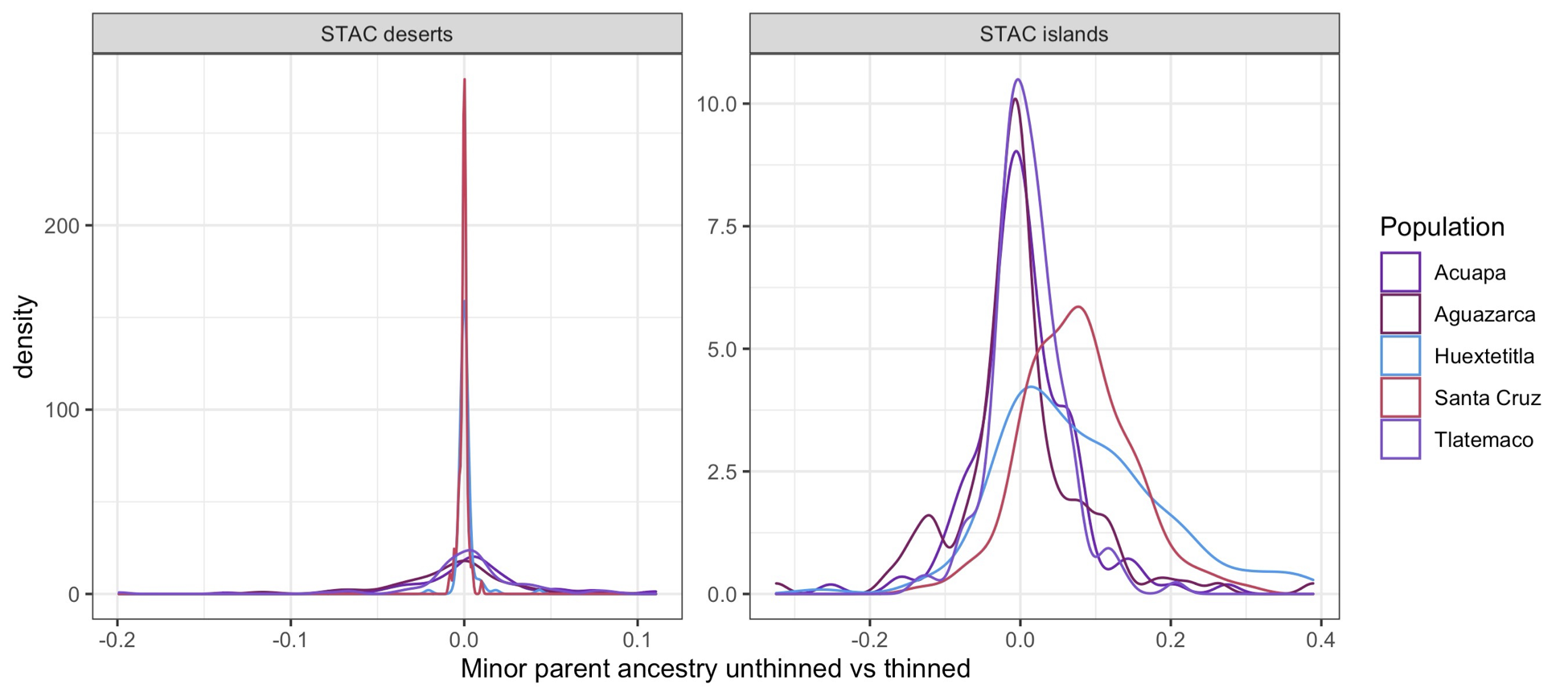

Supplement: S13 Fig — To ensure that minor parent islands and deserts were not generated as an artifact of variation in power to call ancestry along the genome, we re-calculated average ancestry in these regions using ancestry posterior probabilities generated from an input set of ancestry informative markers that were thinned to reduce power differences between different regions of the genome (see Methods; Local ancestry inference in X. birchmanni × X. cortezi hybrids). We found few differences in minor parent ancestry in deserts (A) based on this analysis. We identified more variation in ancestry in minor parent islands in the thinned data (B), and excluded a subset of these islands from further analysis (see Methods). (TIFF) [file pgen.1009914.s033.tiff]

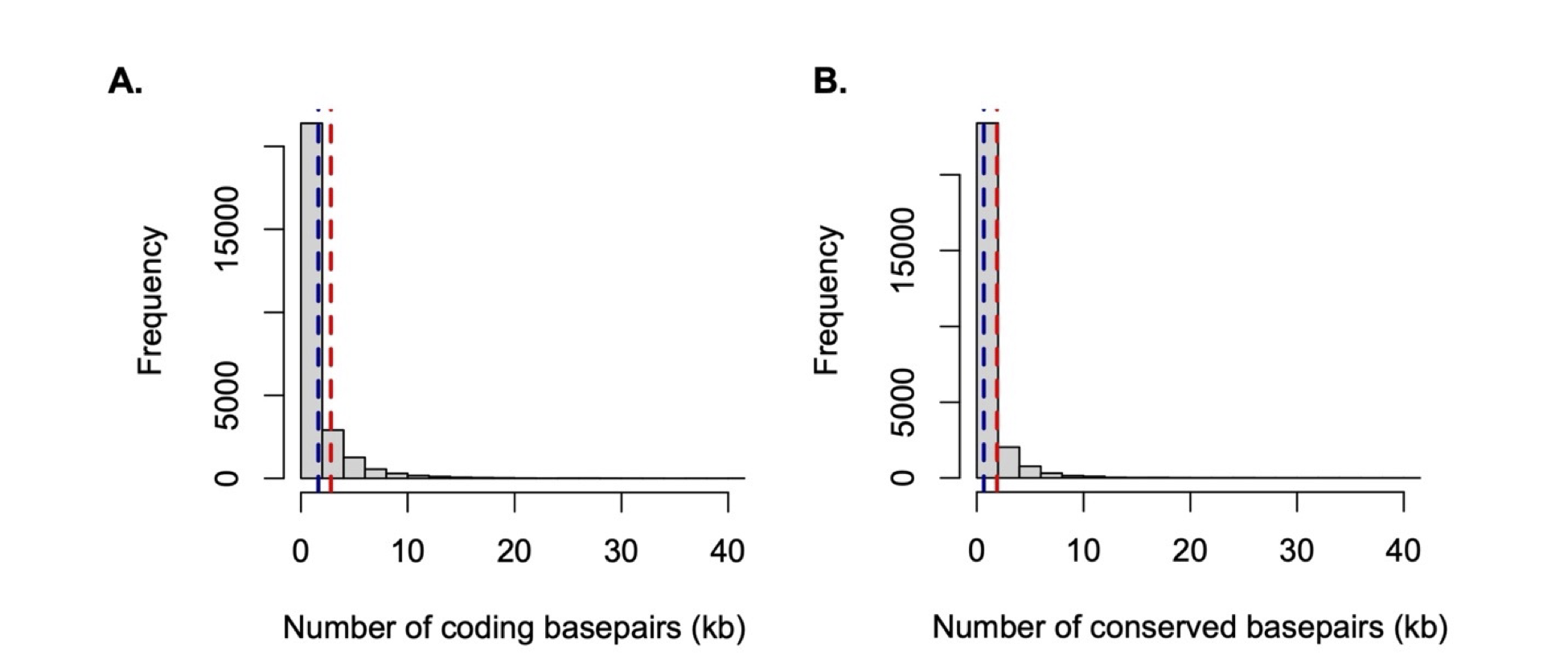

Supplement: S14 Fig — Shared minor parent ancestry deserts and islands do not have an excess of coding (A) or conserved (B) basepairs compared to other regions of the genome that were not shared ancestry outliers. Gray distributions show number of coding and conserved basepairs in each 0.05 cM window across the genome. Red lines show the median number of coding or conserved basepairs in the 0.05 cM window that is the midpoint of the shared minor parent ancestry deserts. Blue lines show the median number of coding or conserved basepairs in the 0.05 cM window that is the midpoint of the shared minor parent ancestry islands. (TIFF) [file pgen.1009914.s034.tiff]

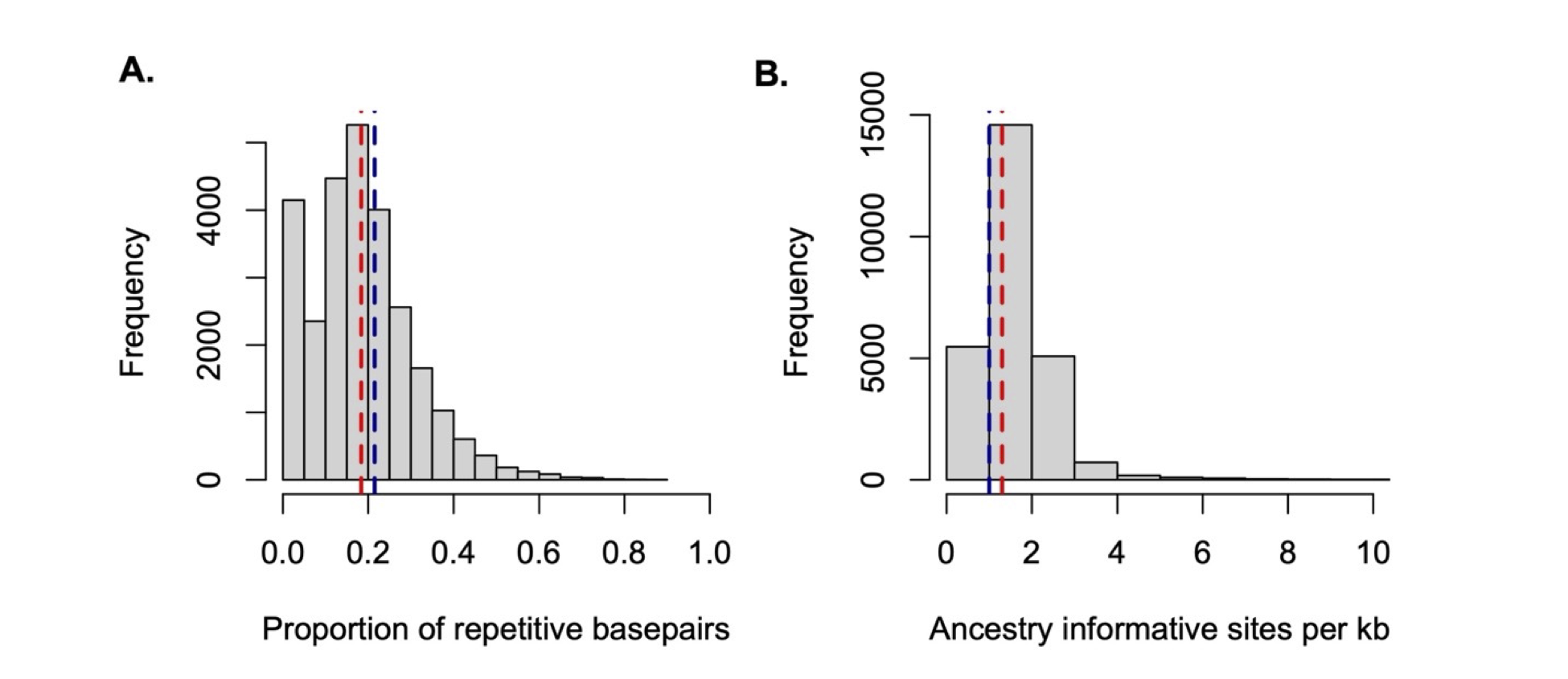

Supplement: S15 Fig — Evaluation of density of repetitive elements (A) and ancestry informative sites (B) in minor parent ancestry deserts and islands relative to the genome-wide background. Distribution in gray shows 0.05 cM windows genome wide, red line shows the median value for the 0.05 cM window that is the midpoint of the shared minor parent ancestry desert, and blue line shows the median value for the 0.05 cM window that is the midpoint of the shared minor parent ancestry islands. (TIFF) [file pgen.1009914.s035.tiff]

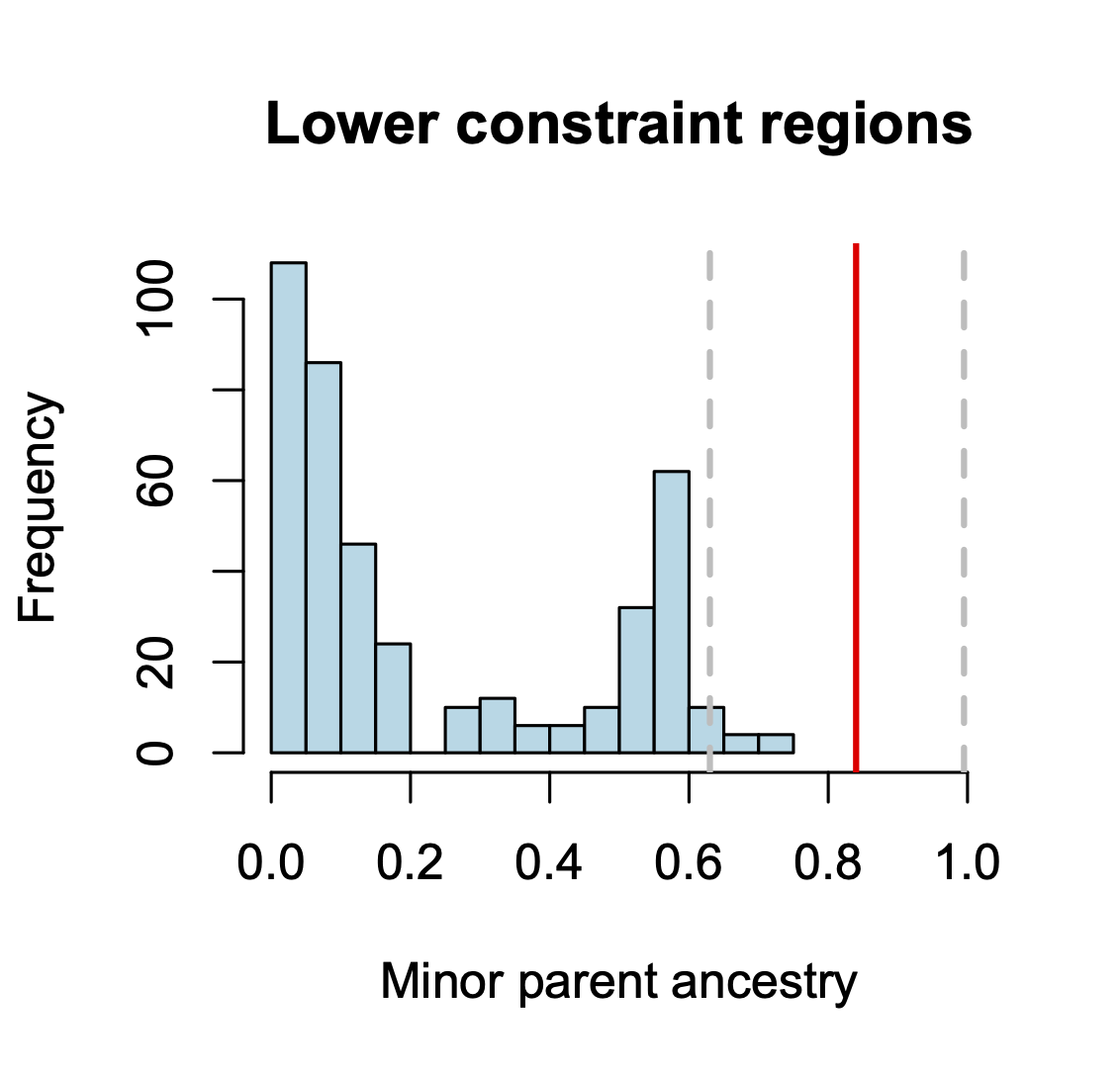

Supplement: S16 Fig — The blue distribution shows minor parent ancestry in the Santa Cruz population in 10 kb windows that are greater than 100 kb from the nearest coding basepair and with an inferred recombination rate in the upper 50% quantile of the genome-wide distribution. The red line shows the average minor parent ancestry in minor parent islands and the gray dashed lines shows the 95% confidence intervals. Thus, in addition to harboring a typical number of coding and conserved basepairs, minor parent islands are still ancestry outliers when compared to regions of the genome expected to have especially low constraint. (TIFF) [file pgen.1009914.s036.tiff]

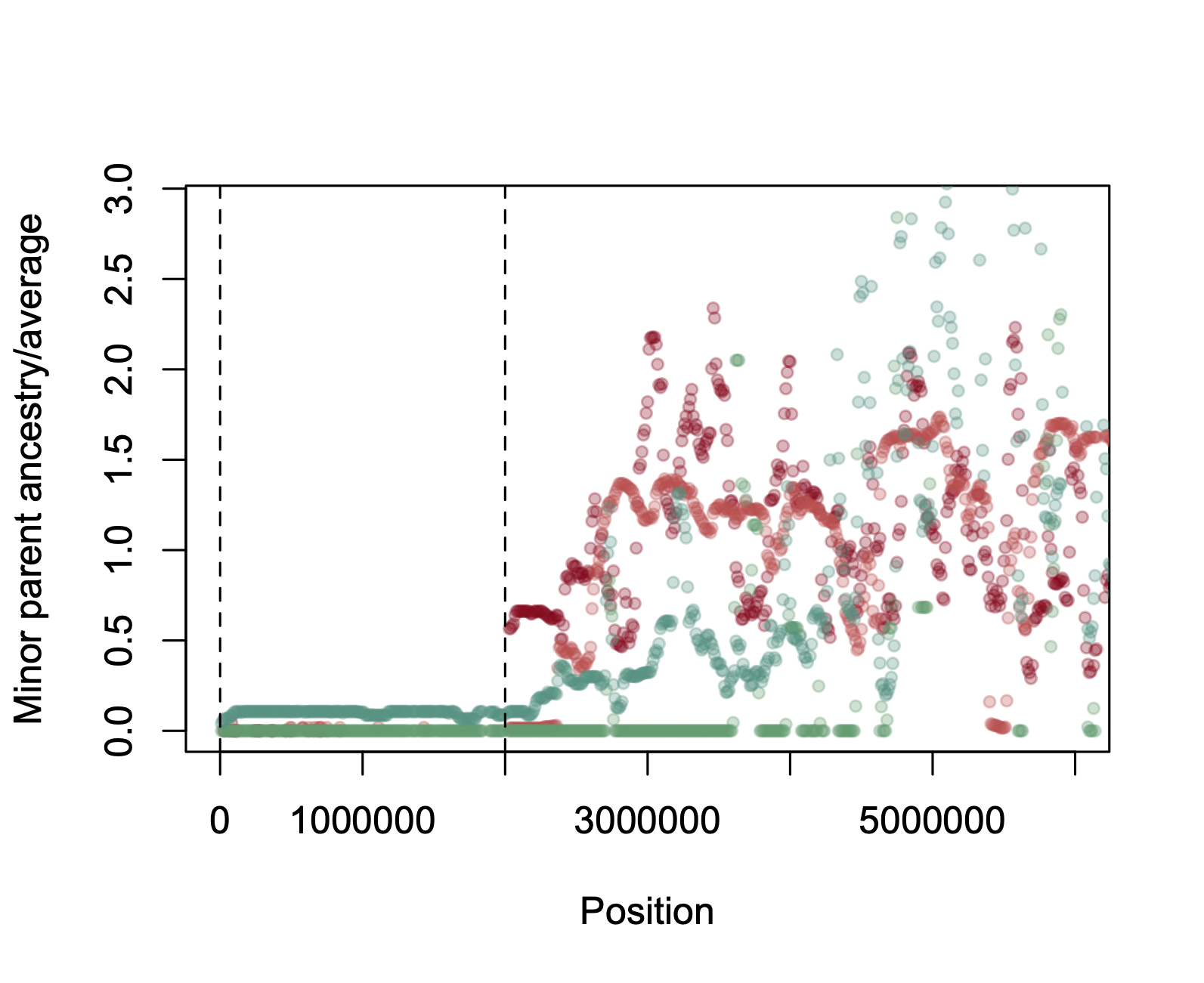

Supplement: S17 Fig — Plotted here is minor parent ancestry in 10 kb windows relative to average minor parent ancestry genome-wide. Green indicates data from X. birchmanni × X. cortezi populations and red indicates data from X. birchmanni × X. malinche populations. (TIFF) [file pgen.1009914.s037.tiff]

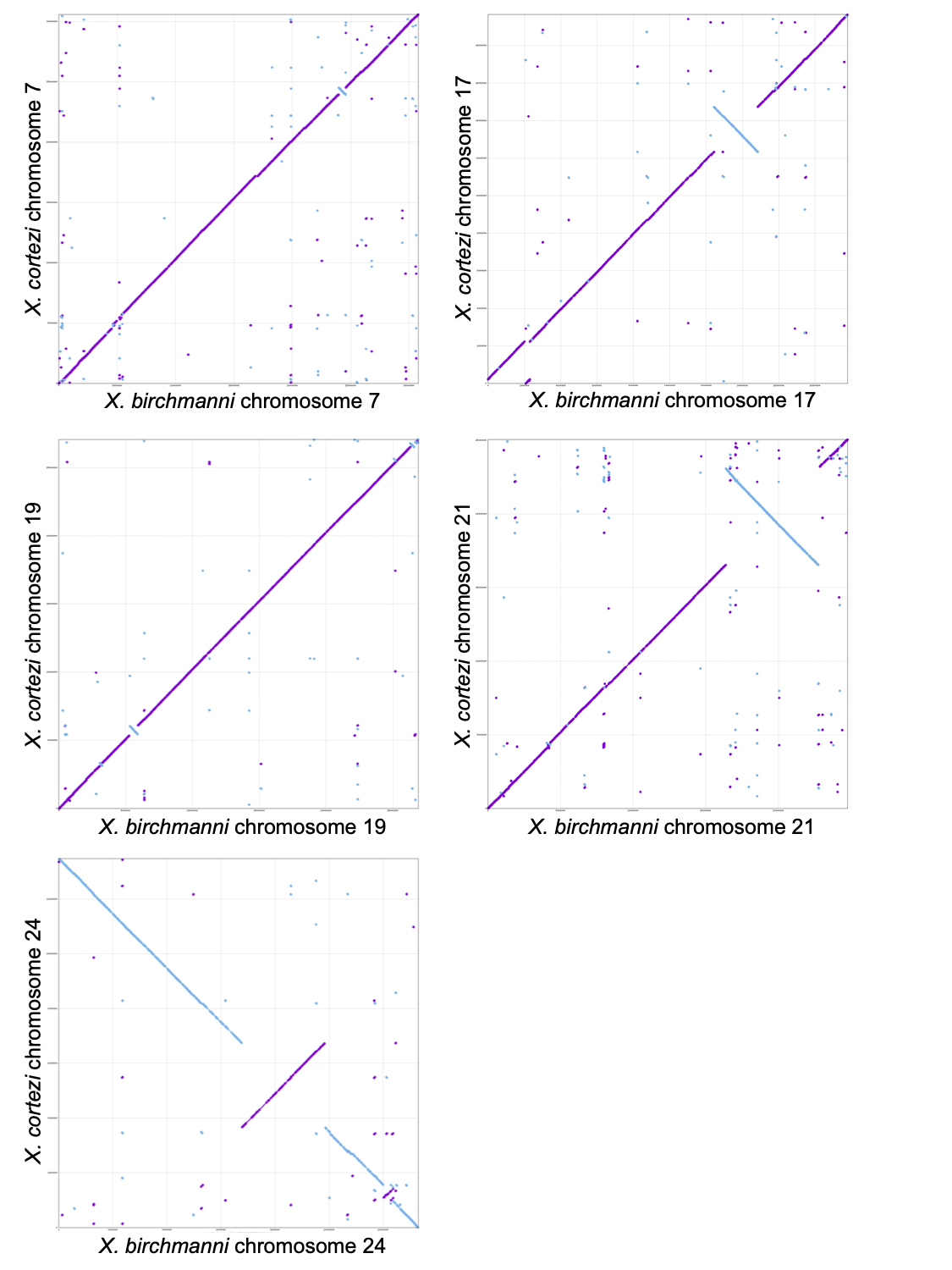

Supplement: S18 Fig — Alignments shown here also include chromosomes where a shared minor parent desert or island was found to overlap with an inversion. (TIFF) [file pgen.1009914.s038.tiff]

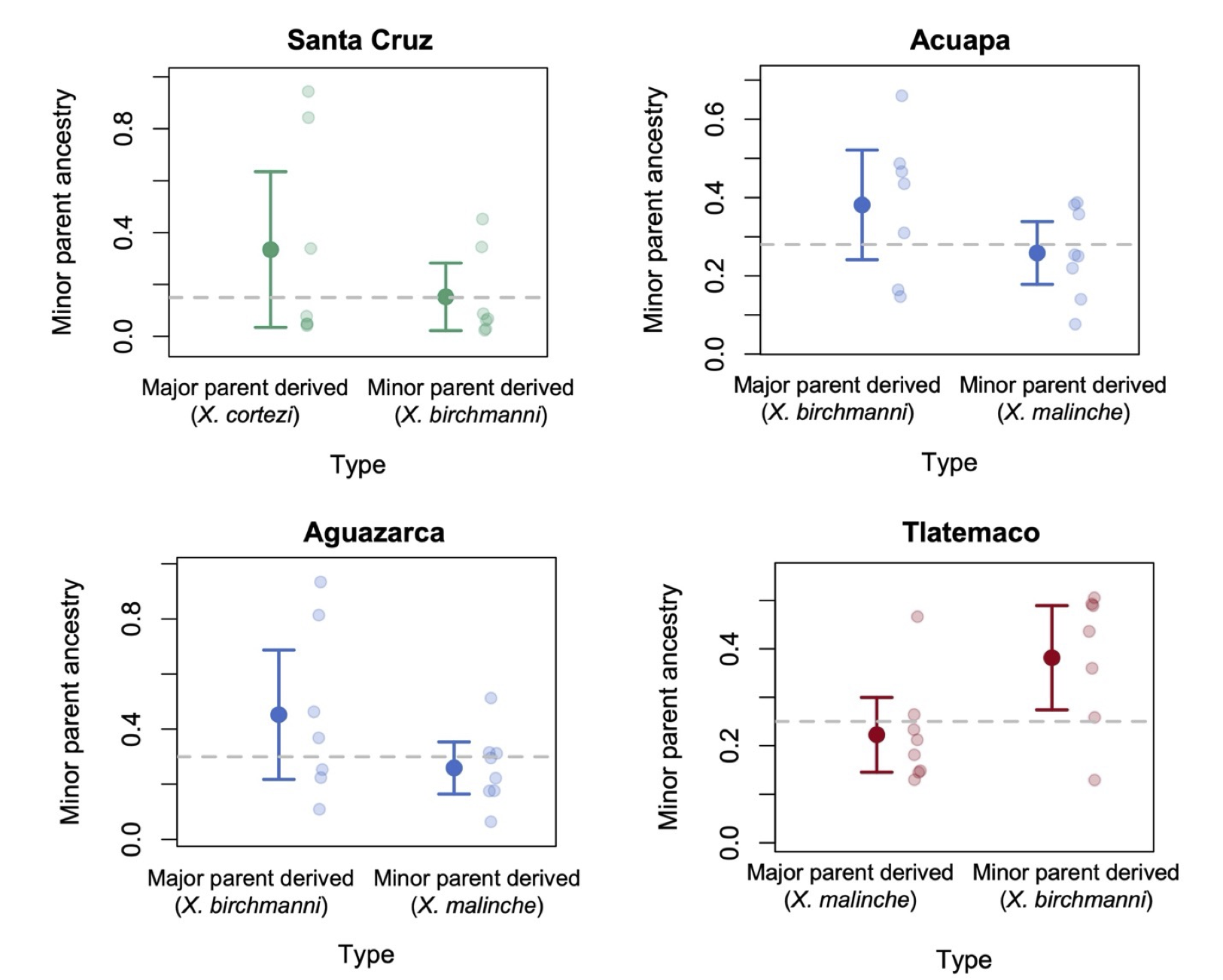

Supplement: S19 Fig — Here we plot minor parent ancestry at inversions that are derived in the major versus minor parent in each independent hybrid population. We find that inversions have unexpectedly high minor parent ancestry regardless of their origin in all hybrid populations, and in several populations inversions derived from the major parent are at unexpectedly low frequencies. Semi-transparent dots show ancestry at individual inversions, solid points and whiskers show the mean ancestry ± 2 standard errors of the mean. Gray line shows average minor parent ancestry in that population genome-wide. (TIFF) [file pgen.1009914.s039.tiff]

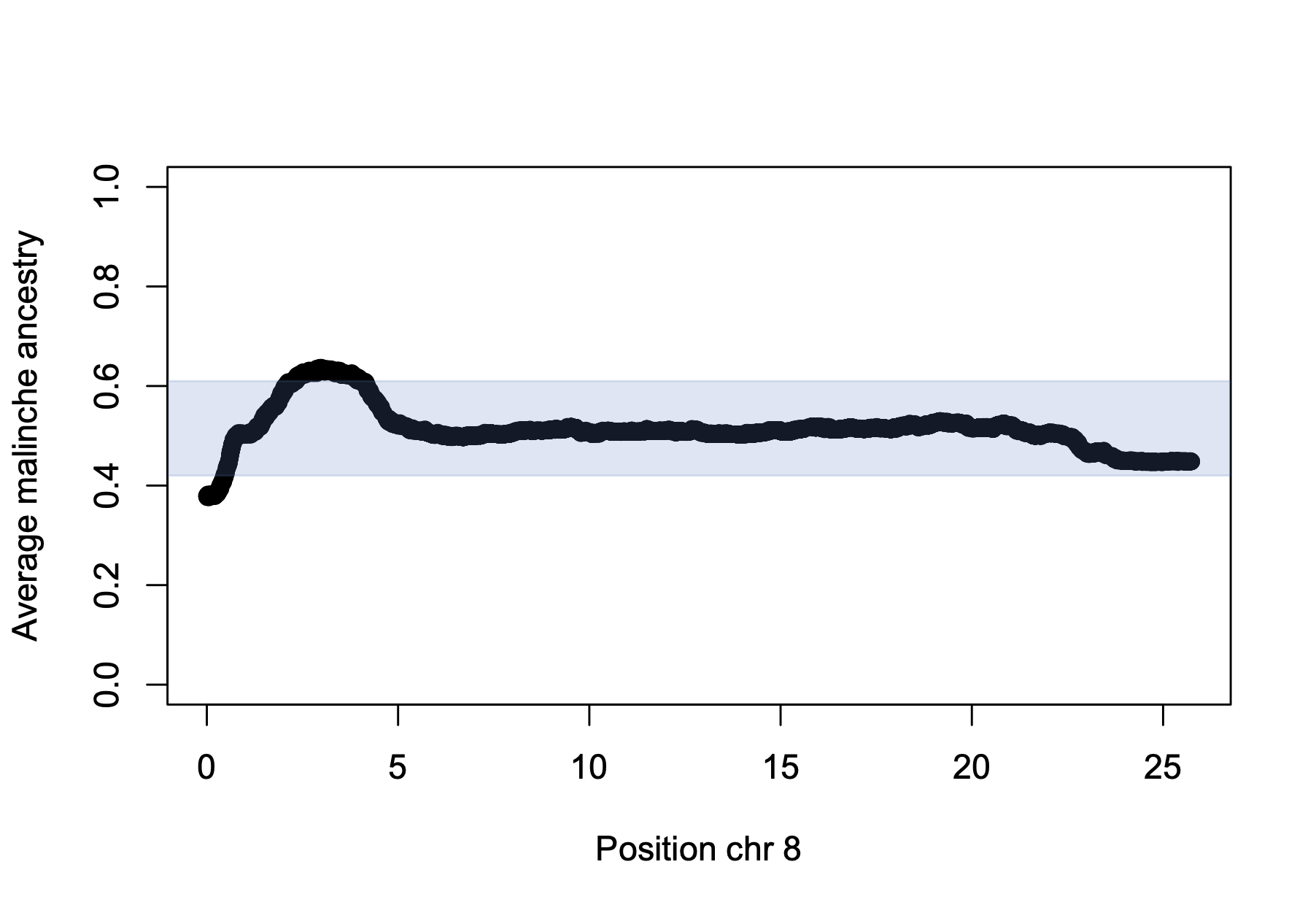

Supplement: S20 Fig — Given the cross design of an F1 intercross we expect 50–50 segregation for parental ancestry types. Indeed, genome-wide average ancestry is 50.3% X. malinche. Plotted here is average ancestry by site along chromosome 8. Chromosome 8 has two regions that fall outside of the 99% confidence intervals for ancestry in the cross (shown by the blue shading). (TIFF) [file pgen.1009914.s040.tiff]

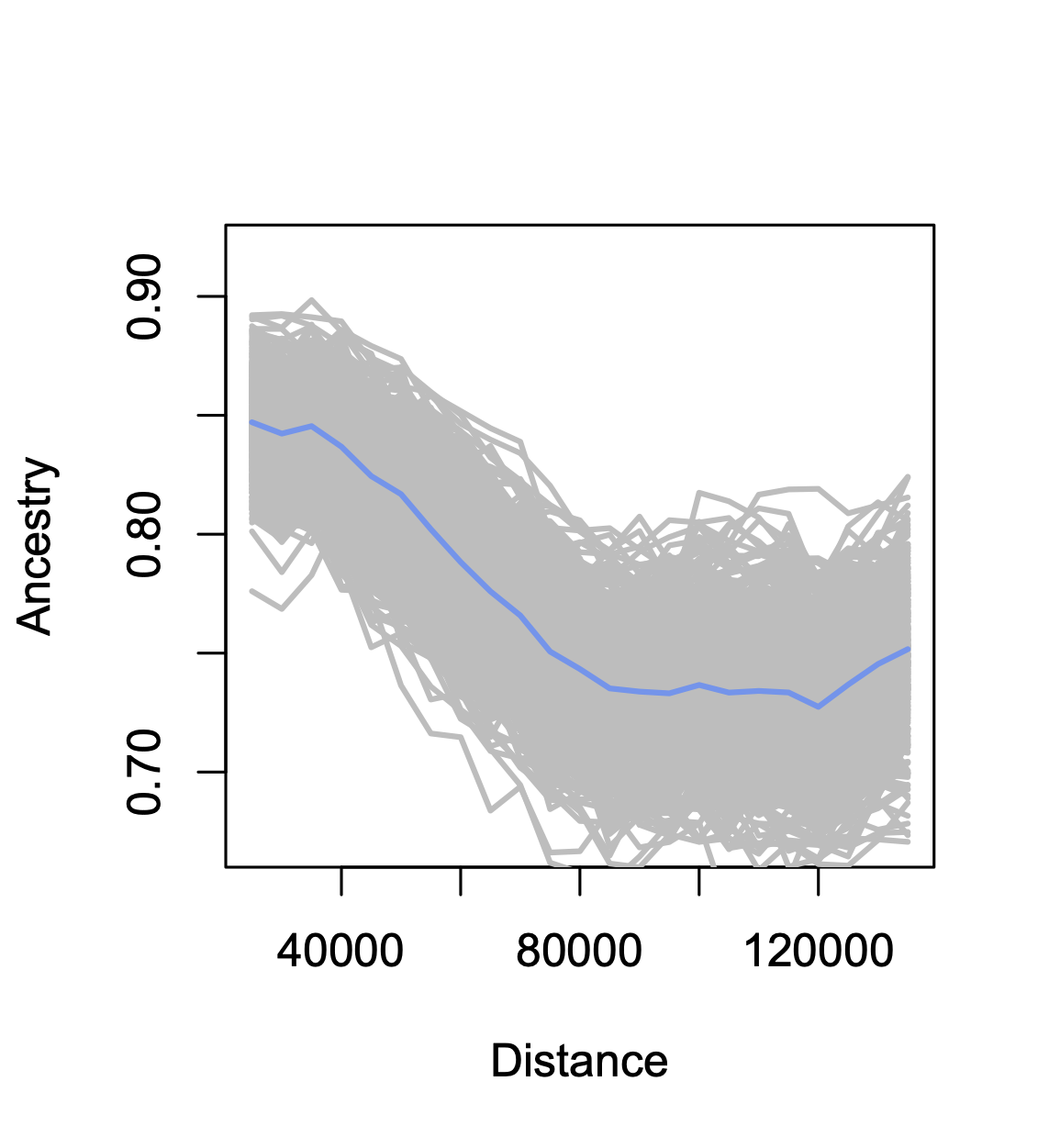

Supplement: S21 Fig — (TIFF) [file pgen.1009914.s041.tiff]

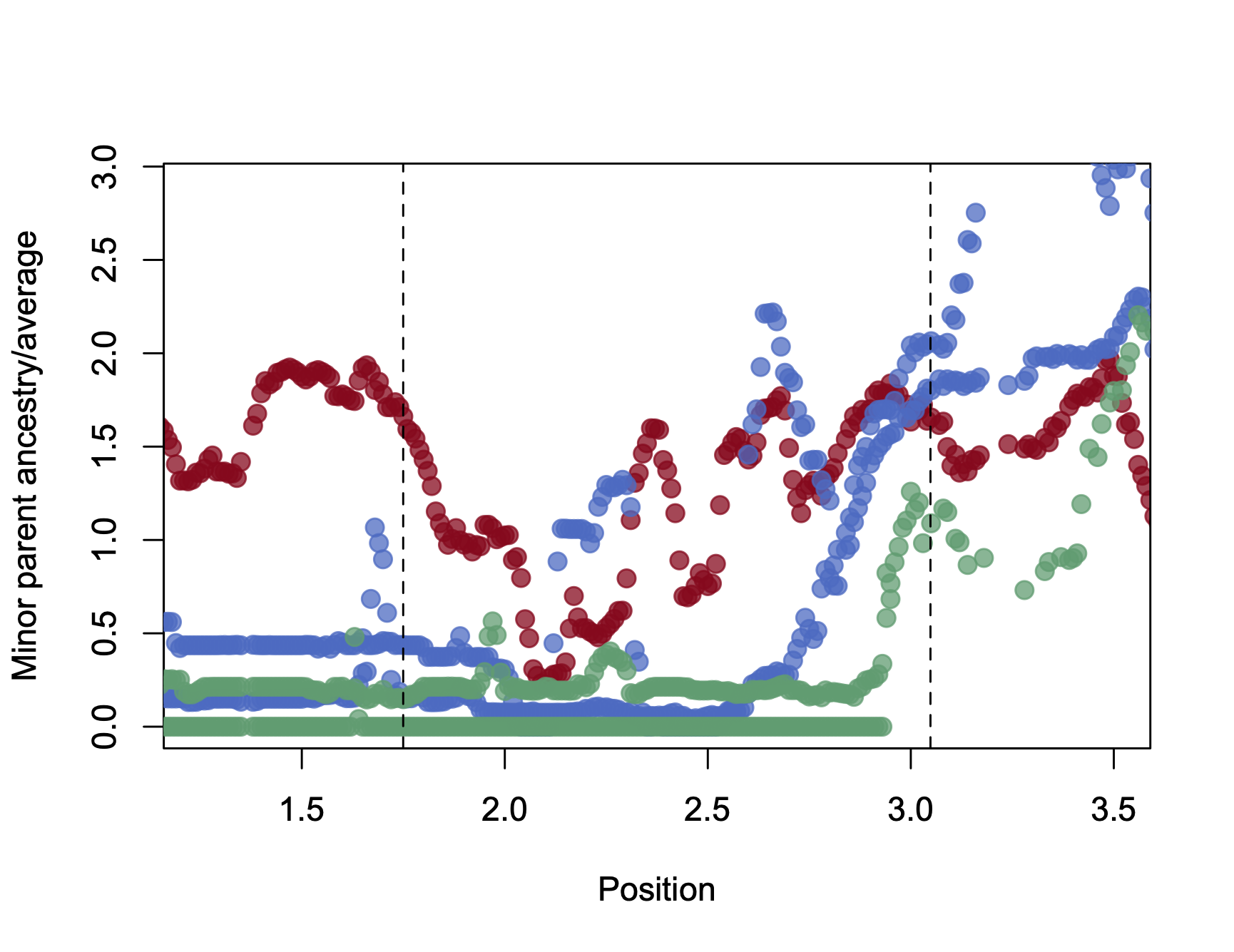

Supplement: S22 Fig — Red–Tlatemaco (malinche × birchmanni), Blue–Acuapa and Aguazarca (birchmanni × malinche), Green–Santa Cruz (birchmanni × cortezi). (TIFF) [file pgen.1009914.s042.tiff]

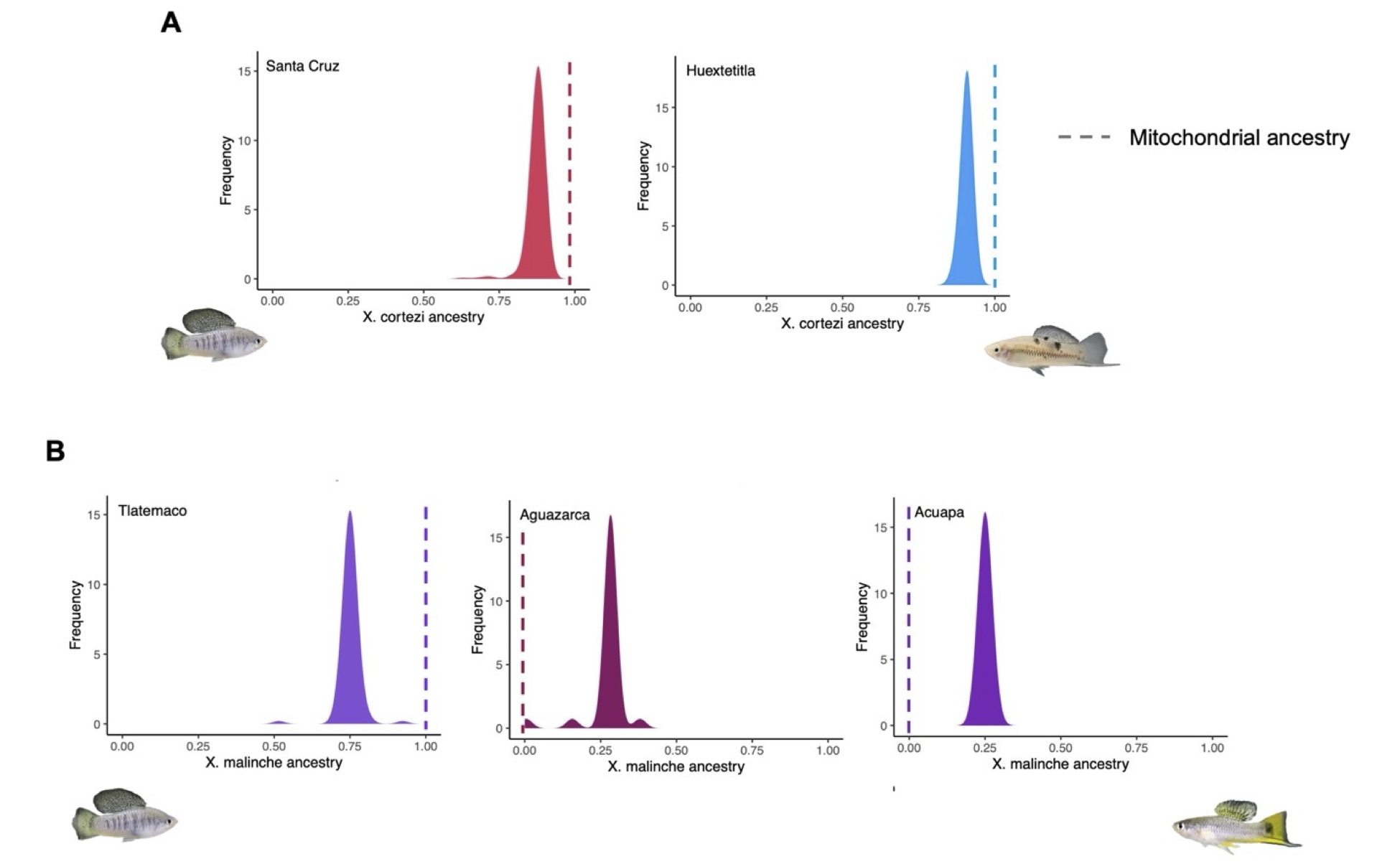

Supplement: S23 Fig — Shown here are the genome-wide ancestry distributions for individuals in X. birchmanni × X. cortezi hybrid populations (A) and X. birchmanni × X. malinche hybrid populations (B). Dotted lines show the ancestry of the mitochondrial haplotype for which the population is fixed. (TIFF) [file pgen.1009914.s043.tiff]

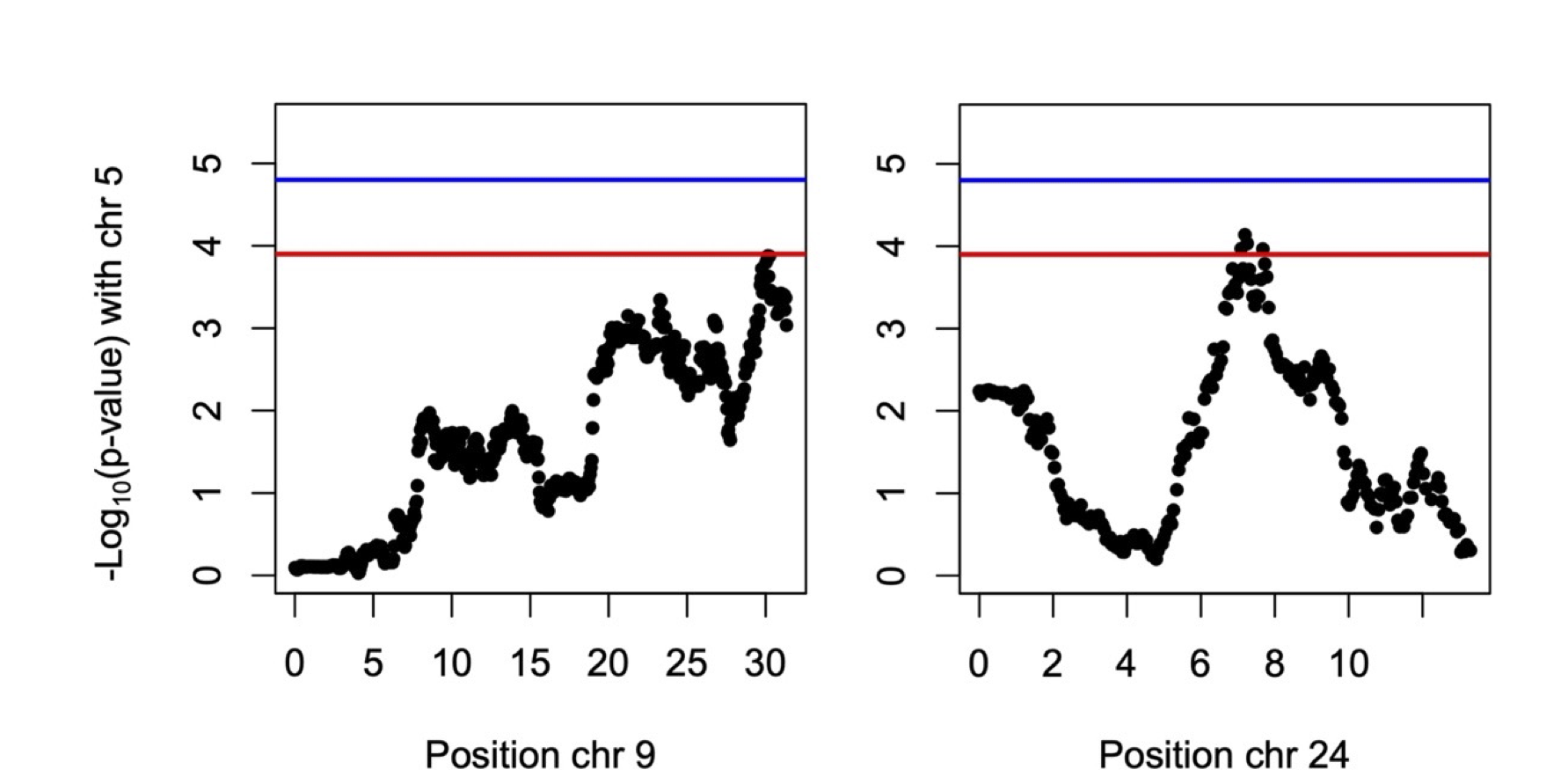

Supplement: S24 Fig — Association between chromosome 5 desert and chromosome 9 and chromosome 24, detected at a FPR of 10% (red line). The FPR 5% threshold is also shown (blue line). Several known gene interactions exist between these three regions: chromosome 24 and chromosome 5—RBM43 and trim25, rnd3b and rasal3, chromosome 9 and chromosome 24—prrx1a and ccnt2b. (TIFF) [file pgen.1009914.s044.tiff]

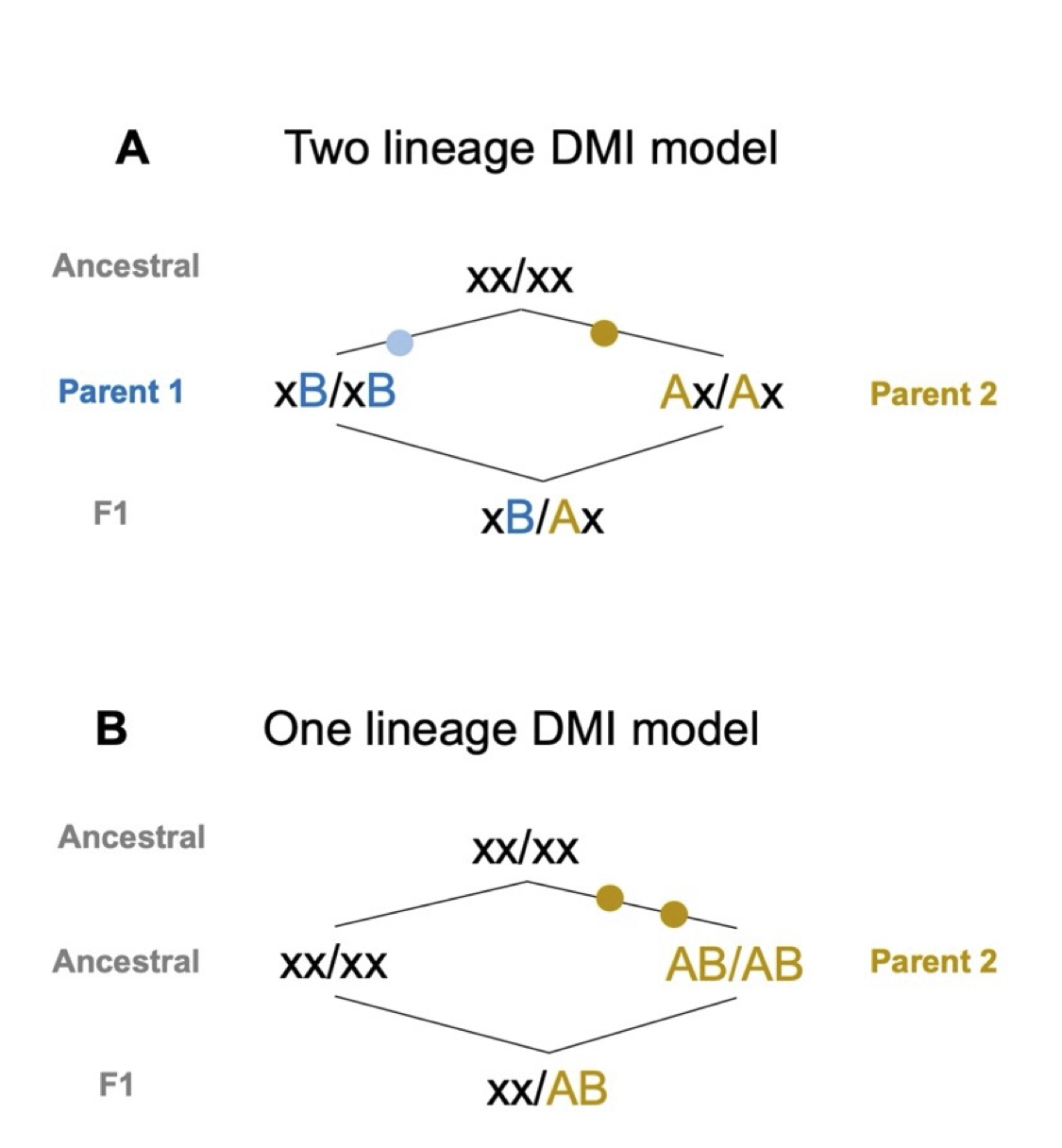

Supplement: S25 Fig — A. As typically depicted, a derived mutation may arise in each lineage (A and B) which has the potential to negatively interact in hybrids. B. DMIs may also arise between the ancestral genotype (denoted as x alleles) and derived alleles that have accumulated on one lineage. This latter scenario may be a possible route through which shared hybrid incompatibilities accumulate between related species, if one lineage has fixed several substitutions and others retain the ancestral genotype. (TIFF) [file pgen.1009914.s045.tiff]

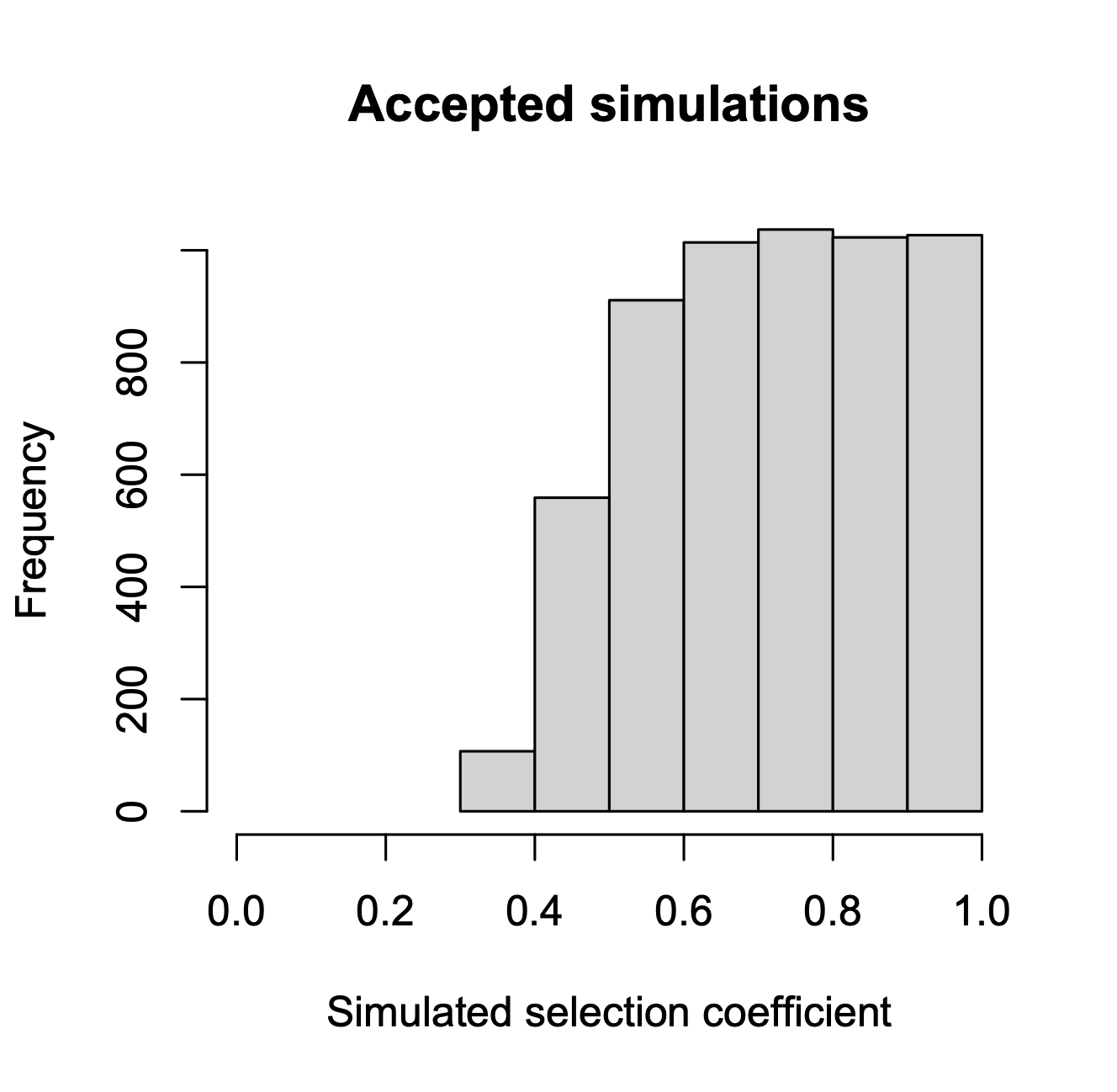

Supplement: S26 Fig — We performed simulations to ask what selection coefficients are consistent with the deviations from expected admixture proportions that we observe at segregation distortion loci. Shown here is the distribution of accepted selection coefficients from simulations (prior s 0–1); see Methods for simulation descriptions. (TIFF) [file pgen.1009914.s046.tiff]

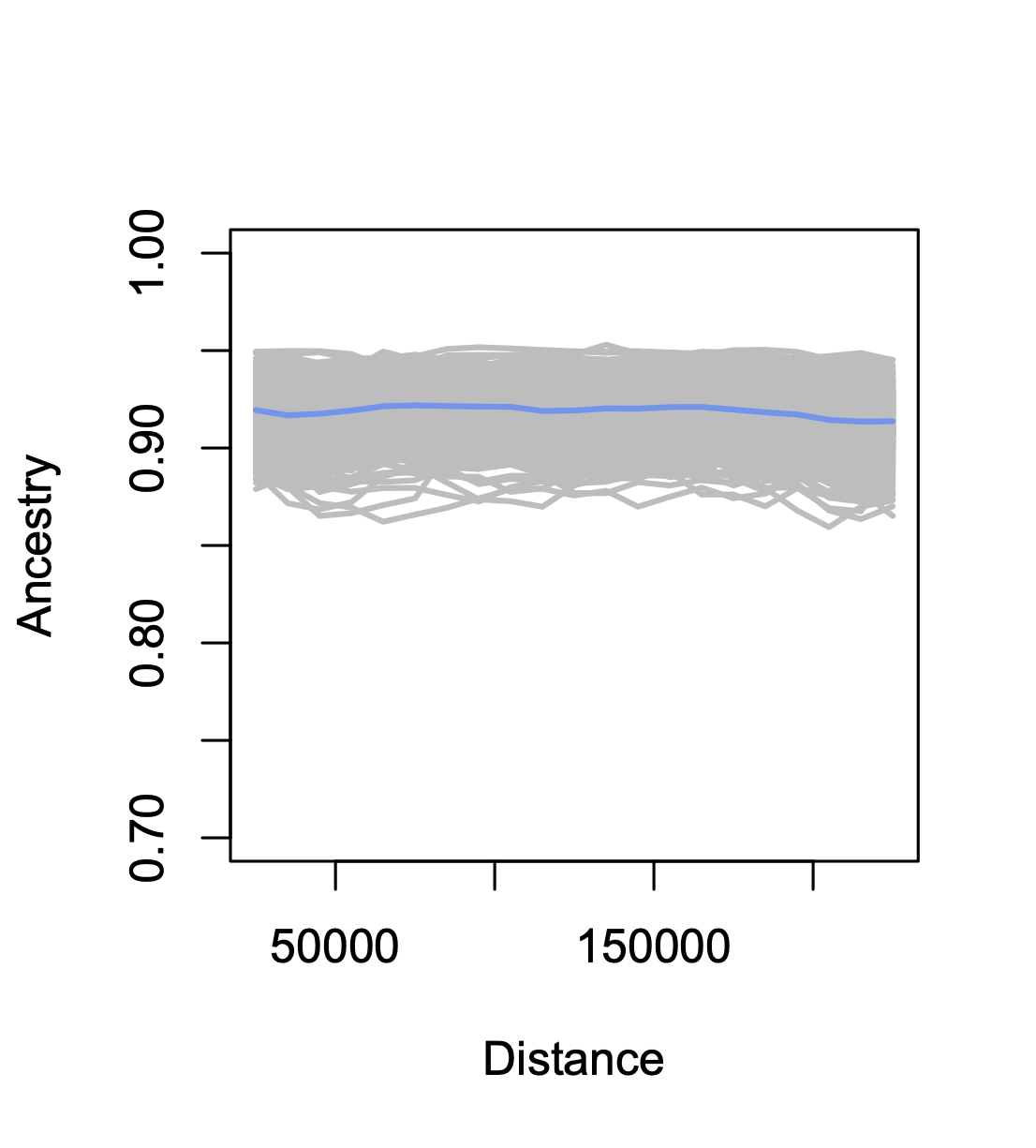

Supplement: S27 Fig — Shown here are the results for the Santa Cruz population as a function of distance to these sites. Gray lines show results of 500 replicates bootstrap resampling the data, blue shows the average across simulations. (TIFF) [file pgen.1009914.s047.tiff]
