# Supplementary material for: Predictability and parallelism in the contemporary evolution of hybrid genomes
Source: PLoS Genet. 2022 Jan 27;18(1):e1009914. doi: 10.1371/journal.pgen.1009914 (PMC8794199; doi:10.1371/journal.pgen.1009914)
Supplement: Attachment — Submitted filename: Langdon_etal_response_to_reviewers.docx [file pgen.1009914.s048.docx]

**Response to Reviewer 1**

Reviewer #1: In this manuscript, Langdon et al. describe and compare patterns of ancestry across multiple admixed populations resulting from hybridization between X. birchmanni and X. cortezi or X. malinche. This results are put in the context of the nature and determinants of genome stabilization following hybridization and what we can learn about the repeatability and predictability of evolution from this. The key results of the paper are (i) that patterns of ancestry are at least somewhat repeatable (more so then expected from neutral evolution), (ii) that repeatability stems in part from selection against minor parent ancestry which interacts with variation in recombination rates across the genome, and (iii) that repeatability also likely reflects the effects of specific incompatibility loci, some of which are identified with some confidence. I found the manuscript clear and interesting overall (i.e., I much enjoyed reading it). I was generally happy with the thoroughness of the analyses, which I think mostly support the authors' claims. With that said, I do have some suggestions for further improving the manuscript.

We thank Reviewer 1 for their recognition of the quality and impact of our findings and for their suggestions for improving the manuscript.

Main comments:

1. My biggest concern relates to the demographic models, specifically, I am not convinced they are generally a good fit for the data and thus that they justify rejecting a neutral demographic model. I do think there is other good evidence in general for rejecting such a hypothesis, my issue is simply with using the demographic models as justification for this. First, it would be useful to know how well one can estimate the model parameters from the summary statistics used. For this, you can treat simulated data sets (one at a time) as the observed data and estimate the (known) parameters for each. I think it is dangerous to use ABC approaches without doing this (and maybe this was done in a previous paper, but if so say so).

We appreciate these comments from reviewer 1 and have made several changes as a result. First, we wanted to note that the demographic parameter space explored here was based largely on previous work (Powell et al. 2021 *Evolution*) but we have revisited this previous work based on the reviewer’s comments.

We have now evaluated how well the ABC approach is working by treating the simulated data as real data, using 5,000 random draws from simulated parameters and summary statistics. These analyses are described in Supporting Information 3. This procedure suggest that our general approach performs well in estimating demographic parameters. Briefly, we found that the true value for the parameters were contained in the 95% quantile of the posterior distribution in >90% of simulations, and the difference between the maximum a posteriori estimate and the true parameter values was centered on zero.

Second, some of the priors are pretty constrained, which is especially important to note in cases where the posterior shifts little from the prior. Also, for some, it probably makes more sense to use a log uniform rather than uniform prior so that it is uniform across orders of magnitude (otherwise almost all of the prior probability will be on larger values).

We appreciate this comment from the reviewer. Constraint on the priors was motivated by our previous work (Powell et al. 2021 *Evolution*) but we have now revised our approach to include a wider range of parameters for initial admixture proportion and hybrid population size, and implemented log uniform sampling for migration rates. In combination with some changes to our implementation of simulations and summary statistics, we now recover well-resolved posterior distributions for all parameters (Fig. S4). Please see Supporting Information 3 for a complete description of these changes.

Thirds, (which relates back to my first point), is it surprising that the demographic model that was fit without information on the variation in ancestry across the genome being used as a summary statistic fails to capture that aspect of the data? What happens if you include this as a summary statistic.

We appreciate this point from the reviewer and have addressed it in the revised manuscript. We have updated our ABC approach to include variation in local ancestry in 250 kb windows as a summary statistic in a subset of analyses. We find a dramatic reduction in the number of simulations accepted (>10X) when this summary statistic is included instead of variation in hybrid index, so we use these simulation results for only a subset of analyses (see Supporting Information 3).

Reassuringly, we see that posterior distributions from simulations using local ancestry variation as a summary statistic closely mirror the results of our main simulations (Fig. S4), except for hybrid population size. Specifically, accepted simulations including local ancestry variation correspond to simulations with very small population sizes – close to the number of individuals sampled from Santa Cruz. Since we have never recaptured a previously fin-clipped individual in either Santa Cruz or Acuapa, we think that these population size estimates are likely unrealistically low compared to census population sizes.

However, as suggested by the reviewer, we used the small number of accepted simulations using this approach to compare simulated variation in local ancestry to local ancestry in the real data (Fig. S5). As expected, variation in local ancestry is higher than in our original simulations (also shown in Fig. S5), but there are still qualitative differences in patterns of fixation and loss compared to the real data. While there may be a neutral parameter space that could capture these other features of the data, we think it is likely that variation in local ancestry in the real data is explained both by drift (captured in these simulations) and by selection (absent from the simulations). We believe that further evidence for this interpretation comes from the fact that in neutral simulations unrealistically small population sizes are required to generate local ancestry variation more consistent with our empirical data. We discuss the implications of these simulation results in more detail in Supporting Information 3, which serve as an important null hypothesis for evaluating predicted ancestry patterns under a range of demographic scenarios in the absence of selection.

2. As far as I can tell, the recombination map used was inferred from patterns of LD in nature and thus reflects the composite parameter capturing recombination and Ne. Assuming this is true, the variation in "recombination" across the genome is really variation in recombination and selection across the genome. This makes it a bit trickier to interpret the correlations between recombination rate and ancestry at the local scales (i.e., across windows). I think this is mostly fine, but is worth acknowledging.

Reviewer 1 raises an important point here, that we had previously attempted to address but now evaluate more fully in the revised manuscript. Specifically, because Ne varies throughout the genome due to differing effects of background selection, so too does our power to infer ρ (4Ne*r). We previously attempted to address this by using what we refer to as a “thinned” LD map, where the input set of SNPs was thinned in regions where SNP density was especially high, to reduce differences in power to infer ρ throughout the genome. We showed that our results were unchanged using an LD map inferred with this approach (see Methods).

While this approach ensures that recombination rates are inferred based on approximately the same number of SNPs for different regions of the genome, it does not completely account for the issue that the reviewer raises, as other features, such as the site frequency spectrum, will differ between regions as a result of background selection. We thus have added analyses that take advantage of a low-resolution crossover map generated from the 943 F_2_ hybrids between *X. birchmanni* and *X. malinche* (total number of ancestry transitions across 24 chromosomes = 34,939). We now show that we reach the same conclusions about the role of recombination rate when relying on the crossover map rather than the LD maps, albeit at larger spatial scales.

Other minor comments:

L170-180, I didn't see where a bottleneck model was considered? From what I can tell the ABC model only specified a single hybrid population size not an initial bottleneck population size followed by a rebound.

The reviewer is correct that we implemented a single population size for simulations rather than a bottleneck followed by a recovery. Because admixture began so recently (see also Powell et al. 2021 *Evolution*) we did not think the timescale or available summary statistics for the real data were sufficient to yield information about two (or more) different population sizes. Future work incorporating information on genetic diversity within ancestry tracts could begin to address this question.

L242, This is subjective, but I don't know that I would call rho values of 0.25 and 0.17 "surprisingly high". Rank correlations are perhaps a bit tricky to think about, but it we treat these like Pearson correlations and then calculate r2 from that, this would suggest that patterns of minor parent ancestry in one species explain ~3% (0.17^2 = 0.0289) to ~6% (0.25^2 = 0.0625) of the variation in patterns of ancestry in the other. I think saying something like that would better convey the results.

We see the reviewer’s point here and have modified our language accordingly.

L298, Probably should change "indicate" to "consistent with".

This change has been made (line 335).

L350, Typo, "cross" to "across".

This change has been made (line 386).

Table 1, Is there a mistake here with the dN/dS ratios or do they really span ~6 orders of magnitude (i.e., 0.001 to 102). IN particular, maybe 102 and 98 should be 1.02 and 0.98 or something like that.

The dN/dS ratios were correct as originally listed but we have updated the table to make the reason for such high dN/dS ratios clear. Specifically, the ratios varied so much because of few or no synonymous substitutions in certain genes. We have now listed these ratios as >99 and made clear the role of the dS denominator in contributing to these large values. We note, however, that these high values are not simply an artifact of low dS. *ndufs5* and *ndufa13* are both unusual in the number of nonsynonymous substitutions between *X. birchmanni* and *X. cortezi* (or *X. malinche*) genome-wide among genes of similar length. Specifically, *ndufs5* falls in the upper 1% tail in the number of nonsynonymous substitutions among genes genome-wide among genes 290-350 basepairs (within 10% of the length of *ndufs5*), and *ndufa13* falls in the upper 2% tail among genes of similar length genome-wide (390 – 480 basepairs, within 10% of the length of *ndufa13*).

L421, Others have considered predictability and repeatability in the context of hybridization and even specifically genome stabilization. See in particular Rieseberg et al. 2003 (10.1126/science.1086949) (which is cited but should be mentioned here and in this specific context) and related work on sunflowers and Chaturvedi et al. 2020 (<https://doi.org/10.1038/s41467-020-15641-x>).

We have added these references (line 459).

Fig 2 B, This should be Spearman rho not Pearson R I think, and what is the Pearson correlation (or why use rank correlations for everything here, consider reporting both).

We have corrected this error (line 1021).

**Response to Reviewer 2**

This is a very nice paper on an amazing study system. The authors examine whether the patterns of genome ancestry in hybrids are repeatable among replicated populations of hybrids from the same parental species and among different sets of parental species. This seems to be high quality and rigorous work. I have only a few comments:

We appreciate Reviewer 2’s suggestions for improvement of the manuscript and thank them for their recognition of the quality and rigor of our work.

The paper is densely written and refers to a lot of supplementary material, which makes it hard to read and follow at some places and a difficult paper to review. Also, it would be useful in the results to detail the method since the method sections comes after the results. Including more details on the assumptions behind the analyses and simulations would also help. For instance, on line 251, the authors say ‘after accounting for shared genomic architecture, we still’ . The accounting for the shared architecture seems a very critical analysis here since the signal left is attributed to selection. It would be useful to know how this accounting was done and how robust it is to all the assumptions that the authors have to put into it.

In response to this comment, we have added abbreviated methods where appropriate (see lines 204-210, 289-296). We have also been more explicit in the main text about how certain analyses were done, such as accounting for shared genomic architecture (lines 266-272).

Figure 2A: I imagine that the windows are non-overlapping windows?

Yes, we have made this clear in the revised manuscript.

Figure 3A: y-axis could be better labeled. A color legend for the grey/colored points would be useful.

We have updated this figure.

In the analyses where there is a relationship between conserved base pairs and ancestry of particular windows. I wonder if the accuracy of the ancestry calling also depends on the level of nucleotide divergence in these windows. Since priors are used to assign the ancestry, a weak signal due to few SNPs for calling the ancestry may be more affected by the priors and favor one parent or the other?

Reviewer 2 raises an important point here, one that we tried to address with certain analyses in the original version of the manuscript and investigate further in the revised manuscript. Specifically, regions of the genome that have many ancestry informative sites might be expected to have fewer conserved basepairs, as such regions by definition are more divergent between species. Since accuracy in local ancestry inference is dependent on the density of ancestry informative sites, if unaccounted for, this could introduce artifacts in our analyses of correlations between ancestry and the density of conserved basepairs.

In the original version of the manuscript, we included one major analysis to account for this issue. Specifically, we attempted to control for variation in power to infer ancestry along the genome by thinning markers in regions where ancestry informative marker density was high (described in Methods, line 634-646). We then re-ran local ancestry inference and confirmed that our inferences about relationships between minor parent ancestry and coding and conserved basepairs were unchanged (see results reported in Table S2).

Based on the reviewer’s comments, we further evaluated this possible technical issue by adding a new analysis. We repeated the analysis of the relationship between conserved basepairs and minor parent ancestry excluding regions of especially high or low ancestry informative site density. Specifically, we removed windows that fell in the upper or lower quantile of ancestry informative site density and repeated the analysis. We found that excluding windows with few or many ancestry informative sites did not impact our results. In all cases we identified a significant negative correlation between minor parent ancestry and the number of conserved basepairs (in analyses of 0.05, 0.1, 0.25 and 0.5 cM windows). These analyses, shown below, suggest that our results are not driven by variation in power to infer ancestry between windows. We report a subset of these results in Table S9.

|  | 0.05 cM | 0.1 cM | 0.25 cM | 0.5 cM |
| --- | --- | --- | --- | --- |
| Santa Cruz | ρ = - 0.1, p<10^-36^ | ρ = - 0.12, p<10^-28^ | ρ = - 0.17, p<10^-22^ | ρ = - 0.2, p<10^-16^ |
| Huextetitla | ρ = - 0.07, p<10^-18^ | ρ = - 0.09, p<10^-16^ | ρ = - 0.13, p<10^-14^ | ρ = - 0.18, p<10^-12^ |

Line 242: Re the correlation of ancestry across the genome. I was wondering if windows are non-overlapping to make sure the correlation tests are valid.

Windows were non-overlapping, this has been clarified throughout the manuscript.

I am not sure I understand the data presented in Table 1. Is the Dn/Ds ratio shown 102? From the data described it seems that there is no synonymous change so a ratio should not be calculated because it would be infinite? It would be useful to discuss more the observations and also the quality of the mapping data in these regions to make sure they are clearly a single-copy gene etc.

We thank the reviewer for pointing this out. We have updated Table 1 to address this comment and now list the ratio as >99. As discussed above, the number of non-synonymous substitutions found in both *ndufs5* and *ndufa13* (independent of the synonymous substitutions) is unusual for genes of their length. We have evaluated these genes in more detail in a companion manuscript (Moran et al. *bioRxiv* 2021).

Line 685: it is not clear what the simulations to examine the correlations of ancestry take into account, for instance the presence of inversions in the parental genomes.

We have added this information to the text. Simulations do not model the presence of parental inversions as this is not straightforward to implement. However, we now report results for the empirical data when these regions are excluded (Table S11).

**Response to Reviewer 3**

This paper presents a very thorough exploration of how admixture landscapes are correlated with various genomic features and between different hybrid swordtail fish populations. The authors examine several hybrid populations with different “major” and “minor” parent species (i.e. the parent that contributes the majority of the gene pool versus the minority of the gene pool) and broadly explore the properties of the regions of the genome that contain less minor parent ancestry than expected by chance. Most of the correlations of minor parent ancestry depletion presented in the paper (e.g. with recombination rate, linkage to conserved base pairs) have been hypothesized in the past and demonstrated in other hybrid systems, but it is valuable to see these patterns corroborated in a set of independent hybrid zones being analyzed in a rigorous and consistent way.

We thank Reviewer 3 for their comments on our manuscript and appreciation of the importance of this work for understanding the drivers of ancestry variation after hybridization.

One of the main findings of the paper are shared deserts of minor parent ancestry among parallel swordtail fish hybrid zones, a result that is reminiscent of the overlap in deserts previously identified between Neanderthal and Denisovan introgression landscapes in humans. I think this result would be strengthened if the authors presented a clearer argument for the claim in line 280 that “sharing of both low and high minor parent ancestry regions exceeded sharing expected by chance (Fig 3A; Methods, Supporting Information 7; p<0.001 by simulation).” I couldn’t figure out how this p value was calculated exactly, or whether it suffers from an error commonly made when computing correlations between time series data. Like a traditional time series, the proportion of minor parent ancestry in each population varies continuously along the genome, which implies that if it happens to be low in the same region of two landscapes at once, it will also be low in some surrounding window, and these datapoints cannot be regarded as independent samples due to the continuity of the data. When quantifying the correlation between minor parent ancestry deserts, and especially when assigning it a p value, the authors should take care to avoid this issue (described in more detail here <https://academic.oup.com/mbe/article/23/5/911/1058377>).

Reviewer 3 highlights an important and often overlooked issue in evolutionary genomics. Like other population genomic parameters, local ancestry is autocorrelated along the genome. In the case of summaries of local ancestry in young hybrid populations such as these, this autocorrelation can extend for long physical distances because of the distances over which admixture LD decays. In the case of these *Xiphophorus* populations, admixture LD decays over ~500 kb to 1 Mb, depending on the population (Schumer et al. 2018, Powell et al. 2020, Powell et al. 2021).

In the original version of the manuscript we used two approaches to evaluate the probability that minor parent ancestry deserts or islands were shared across independent hybrid populations by chance. The simplest analysis, which randomly samples windows from pairs of populations (described in the Methods) breaks up autocorrelation in ancestry by sampling in a way that is blind to the spatial structure of the genome. While this approach has the advantage of being simple and easily understandably, it suffers from the issue that reviewer 3 outlines. We also performed an analysis more carefully matching the architecture of shared minor parent deserts and islands (local recombination rate and coding density; Supporting Information 8).

Based on the reviewer’s comments, we have now included a new permutation-based approach that preserves local ancestry structure (see Supporting Information 8). In this approach, we shifted the labels of windows in 12.5 cM blocks in a series of simulations, and asked how often minor parent ancestry deserts or islands overlapped in these permuted datasets by chance. By shifting labels in a block-wise fashion, we can preserve local ancestry structure in the simulated datasets and at the same time ask how likely overlap in local ancestry between populations is by chance (since overlap in the permuted datasets cannot be attributed to selection). We have modified the text to include the results of this second approach, from which results for minor parent deserts are qualitatively similar, but results for minor parent islands are less convincing (Fig. S12).

Based on reviewer 3’s comments, we also evaluated evidence for autocorrelation in minor parent ancestry among deserts and islands that occur on the same chromosome, following Hahn 2006. We focused this analysis on the Santa Cruz hybrid population, where we first identify minor parent ancestry deserts and islands. This set of deserts and islands was used in further analysis of cross-population sharing. We found weak evidence for autocorrelation in ancestry between deserts and islands identified in the Santa Cruz hybrid population. We summarize these results in Supporting Information 8.

From reading supporting information 7, I think this claim of minor parent desert overlap is based on a comparison to a null consisting of simulated populations that were each picked to have negative selection against minor parent ancestry at 1 random site. This seems much too simple to reproduce features like the correlation of recombination rate with minor parent ancestry, since this would require selection against minor parent ancestry at much more than 1 site per genome. Later on in the manuscript the authors clarify that the minor parent ancestry deserts are identified in a way that corrects for recombination rate, and they also perform a post-hoc test showing that exon density can’t account for the results, but I think the simulation they initially compare to is enough of a straw man that it would be more useful to just cite these latter results rather that cite a p-value based on a comparison to genomes where selection acts on just 1 site each.

We apologize that this was unclear in the original manuscript and have now edited it to improve clarity (lines 280-331). The simulations described in Supporting Information 7 were an analysis of power to detect shared ancestry deserts at different selection coefficients. These simulations were not used as a null model for the expected number of shared ancestry deserts by chance in the real data precisely for the reason that the reviewer highlights: simulations with a single selected site will not be of sufficient complexity to reproduce genome-wide features in the real data.

Instead, as discussed above, we use several approaches to evaluate the likelihood of shared ancestry deserts by chance, using the observed local ancestry variation in the empirical data to generate our null expectations. We hope this is clearer in the revised manuscript.

Although this is not essential, I also think the manuscript could be improved by adding more analyses to the Tlatemaco population, which is noted in a few cases to be an outlier. The authors hypothesize that Tlatemaco behaves differently than the others because the minor parent has higher effective population size than the major parent, which could mean that selection for lower genetic load works in opposition to selection to minimize hybrid incompatibilities. Is this hypothesis testable by digging more into where the Tlatemaco landscape exhibits similarities and differences to the other birchmanni x malinche populations? For example, does Tlatemaco display the expected behavior near segregation distorters, which probably aren’t too sensitive to effective population size? What about the other candidate incompatibility loci? Are genes under the most purifying selection like housekeeping genes the ones where this population might display evidence for selection against the major parent ancestry due to genetic load?

We agree with Reviewer 3 that this is an interesting area of inquiry, and is something we have evaluated to some extent in past work (specifically in Schumer et al. 2018 *Science*). We do see that Tlatemaco is less similar in patterns of local ancestry to other *X. birchmanni* x *X. malinche* hybrid populations. As discussed in the manuscript, this may be attributable to differences in effective population sizes of the major parent (*X. malinche* in the case of Tlatemaco vs *X. birchmanni* in the others), but past work has indicated that the Tlatemaco population is also likely older than the Acuapa and Aguazarca hybrid populations.

We do see that minor parent ancestry is reduced in Tlatemaco around segregation distorters, which, as the reviewer points out, are not expected to be sensitive to effective population size since they are inferred to be under strong selection (Fig. S30). We also see reduced minor parent ancestry in Tlatemaco hybrids around other putative DMI loci mapped between *X. birchmanni* x *X. malinche* (see Schumer et al. 2018 *Science*).

We have investigated the reviewer’s final question in the context of previous work comparing *X. birchmanni* x *X. malinche* hybrid populations. We showed that *X. malinche* genomes harbor an excess of derived nonsynonymous sites compared to *X. birchmanni* (Schumer et al. 2018 *Science*, Supporting Information 1.3). Interestingly, we did not see evidence for significantly lower *X. malinche* ancestry around derived nonsynonymous sites in the Tlatemaco hybrid population, as might be expected under a load hypothesis.

Together, these results underscore that load may play an important role in explaining local ancestry differences between Tlatemaco and other *X. birchmanni* x *X. malinche* and *X. birchmanni* x *X. cortezi* hybrid populations. We view a more thorough investigating of these dynamics as outside the scope of the current manuscript but do think this will be an exciting direction for future work.

Minor comment:
“principal components” is misspelled throughout supporting information section 2

This error has been corrected.
